# A network-based trans-omics approach for predicting synergistic drug combinations

**DOI:** 10.1038/s43856-024-00571-2

**Published:** 2024-07-29

**Authors:** Midori Iida, Yurika Kuniki, Kenta Yagi, Mitsuhiro Goda, Satoko Namba, Jun-ichi Takeshita, Ryusuke Sawada, Michio Iwata, Yoshito Zamami, Keisuke Ishizawa, Yoshihiro Yamanishi

**Affiliations:** 1 Department of Physics and Information Technology, Kyushu Institute of Technology, Iizuka, Fukuoka Japan; 2 Department of Bioscience and Bioinformatics, Kyushu Institute of Technology, Iizuka, Fukuoka Japan; 3 Department of Clinical Pharmacology and Therapeutics, Tokushima University Graduate School of Biomedical Sciences, Tokushima, Japan; 4 Clinical Research Center for Developmental Therapeutics, Tokushima University Hospital, Tokushima, Japan; 5 Department of Pharmacy, Tokushima University Hospital, Tokushima, Japan; 6 Department of Complex Systems Science, Graduate School of Informatics, Nagoya University, Chikusa, Nagoya, Aichi Japan; 7 Research Institute of Science for Safety and Sustainability, National Institute of Advanced Industrial Science and Technology (AIST), Tsukuba, Ibaraki Japan; 8 Department of Pharmacology, Okayama University Graduate School of Medicine, Dentistry, and Pharmaceutical Sciences, Okayama, Okayama, Japan; 9 Department of Pharmacy, Okayama University Hospital, Kita-ku, Okayama, Japan

**Keywords:** Virtual drug screening, Virtual screening, Chronic myeloid leukaemia

## Abstract

**Background:**

Combination therapy can offer greater efficacy on medical treatments. However, the discovery of synergistic drug combinations is challenging. We propose a novel computational method, SyndrumNET, to predict synergistic drug combinations by network propagation with trans-omics analyses.

**Methods:**

The prediction is based on the topological relationship, network-based proximity, and transcriptional correlation between diseases and drugs. SyndrumNET was applied to analyzing six diseases including asthma, diabetes, hypertension, colorectal cancer, acute myeloid leukemia (AML), and chronic myeloid leukemia (CML).

**Results:**

Here we show that SyndrumNET outperforms the previous methods in terms of high accuracy. We perform in vitro cell survival assays to validate our prediction for CML. Of the top 17 predicted drug pairs, 14 drug pairs successfully exhibits synergistic anticancer effects. Our mode-of-action analysis also reveals that the drug synergy of the top predicted combination of capsaicin and mitoxantrone is due to the complementary regulation of 12 pathways, including the Rap1 signaling pathway.

**Conclusions:**

The proposed method is expected to be useful for discovering synergistic drug combinations for various complex diseases.

## Plain Language Summary

Adding drug treatments together can sometimes produce better results for patients. We introduced a new computer-based method called SyndrumNET, designed to identify effective drug combinations for treating diseases. The method uses data about how diseases and drugs interact at a molecular level to predict which drugs work well together. Tested on six different diseases, such as asthma and different types of cancer, SyndrumNET proved to be more accurate than previous approaches. For example, most of the drug combinations predicted by SyndrumNET to rank highly have shown better combination effects on leukemia cells. This method also helped understand why certain drug combinations work better by analyzing their effects on cellular pathways. The findings suggest that SyndrumNET could be a valuable tool in developing more effective treatment for various complex diseases.

## Abstract (web summary)

Iida et al. predict synergistic drug combinations using a computational method termed SyndrumNET. Validation of predictions in chronic myeloid leukemia using in vitro cell survival assays reveal synergistic anticancer effects in 14 of 17 top predicted drug pairings.

## Introduction

Combination therapy, which is a treatment modality that combines two or more drugs, can offer greater efficacy or lower individual drug dosages compared with monotherapy^1^. Its effectiveness has been recognized for various complex diseases, such as cancers, hypertension, cardiovascular, neurological, and autoimmune disorders^2,3^. The number of drug combinations approved by the US Food and Drug Administration (FDA) has continuously increased since the first approval of co-administered drugs in the 1940s^4^; however, determining synergistic drug combinations is very challenging, particularly in heterogenous diseases. There are more than 13,000 drugs approved for human use by FDA^5^; thus, the number of possible drug pairs is approximately 85 million. Conducting clinical trials for all possible drug combinations is impractical. Thus, there is a strong need for methods to facilitate the identification of synergistic drug combinations for various diseases.

A variety of computational methods have been developed for predicting synergistic drug combinations^6^. A popular approach is to use supervised learning. For example, pharmacological features (e.g., target proteins, efficacy classes) enriched in approved drug combinations were extracted and new drug combinations associated with the pharmacological features were searched^7^. A sparsity-induced classifier with tensor-based representations of pharmacological features was proposed^8^. A machine learning model using an ensemble of weak predictive models was applied for the dataset in the AstraZeneca-Sanger Drug Combination Prediction DREAM Challenge^9^. A deep learning-based method with structural features of compounds was proposed for COVID-19 and the importance of the structural characteristics of the drugs was determined^10^. However, these supervised learning methods require the prior information on known synergistic drug combinations as a learning dataset to construct predictive models and their performance depends heavily on the quality and quantity of the learning dataset. The number of diseases for which sufficient information on synergistic drug combinations is very limited. For most diseases, synergistic drug combinations remain unknown.

Unsupervised learning is a more practical and realistic approach for predicting synergistic drug combinations because they can be applied to any disease without prior knowledge of synergistic drug combinations^11–13^. For example, a transcriptome-based approach was proposed to predict synergistic drug combinations for glioblastoma (GBM), where the drug was assumed to restore the disease-specific gene expression pattern. For a fixed drug, other drug partners were searched using the inverse correlation between the disease-specific transcriptional expression signatures and drug response gene expression signatures^11^; however, a general framework for any drug pair would be more practical. In addition, the gene expression signatures of diseases and its approved drugs are not always inversely correlated^14^. A network-based approach was proposed to predict synergistic drug combinations for hypertension and cancers based on the relationship between drug target genes and disease genes in the comprehensive molecular interaction network^13^. However, this approach is only applicable to drugs with known targets and it does not take into account the dynamic changes in the cells or organisms associated with drug treatment^15,16^. The therapeutic effect is not only determined by the network-based relationships between diseases and drugs. These previous unsupervised approaches are based on single omics data representing a few biological aspects. Diseases result from the disruption of many biological processes; thus, the integrative use of multi-omics data should contribute to the enhancement of the prediction accuracy of synergistic drug combination.

In this study, we propose a novel computational method, which we call SyndrumNET, to predict synergistic drug combinations by network propagation with trans-omics analyses. The prediction is based on multi-omics data such as genome, transcriptome, interactome, and diseasome data. We demonstrated the usefulness of the proposed method on the prediction of synergistic drug combinations for six diseases: acute myeloid leukemia (AML), chronic myeloid leukemia (CML), colorectal cancer, asthma, type II diabetes, and hypertension. We validated the prediction result for CML through in vitro experiments and identified the underlying mode-of-action of the synergistic effects of the drug combination at the pathway level by microarray analysis.

## Methods

### Construction of the human molecular interaction network

Human molecular interactions were constructed from seven databases (Supplementary Data S1): (i) Yeast-two-hybrid high-throughput datasets were retrieved from the yeast two-hybrid database (HuRI)^17^ (accessed on March 2, 2020), (ii) Protein complexes were retrieved from the CORUM database^18^ (accessed on September 3, 2018), (iii) Kinase–substrate pairs were retrieved from the PhosphositePlus database^19^ (accessed on September 7, 2018), (iv) Metabolic enzyme-coupled interactions were retrieved from the KEGG Rpair database^20^ (accessed on March 12, 2016), (v) Signaling interactions were retrieved from the Signalink v.2.0 database^21^ (accessed on December 3, 2018), (vi) Innate immune response interactions were retrieved from the InnateDB database^22^ (accessed on June 2, 2018), (vii) 3D structurally resolved protein-protein interactions were retrieved from the Instruct database^23^ (accessed on March 3, 2020). We used molecular interactions with biological annotations. We did not include interactions extracted from gene expression data or evolutionary considerations. All interactions from these databases were combined, and the union yielded a network of 13,524 proteins and 311,888 interactions (Supplementary Data S1). Duplicated interactions were excluded using the simplify function in the igraph library (1.2.6) of R. The giant component was used as a human molecular interaction network, and it consisted of 235,123 interactions involving 13,377 proteins (Supplementary Data S2).

This newly established human molecule interaction network offers two advantages. First, it facilitates easy comparison with prior work on network-based drug combination prediction methods. Certain steps in our proposed method align with the findings made by Cheng et al.^13^. To enhance comparability in predictive performance, we adhered to the network creation procedure outlined in the previous work. Second, the network allows for the selection of experimentally validated interaction types. In this study, our focus was solely on molecular interactions with biological annotations (e.g., physical interactions, phosphorylation and substrate–enzyme associations). This emphasis has the potential to enhance the reliability of the network.

### Construction of disease-specific gene expression profiles

The CREEDS database provides gene expression signatures for 79 diseases with 14,804 genes^24^, derived from transcriptome data registered in Gene Expression Omnibus (GEO)^25^. We retrieved disease-specific gene expression signatures of AML, CML, colorectal cancer, asthma, and type 2 diabetes from CREEDS^24^ (accessed on April 22, 2020).

CREEDS lacked the gene expression signature for hypertension. Thus, we constructed the gene expression signature for hypertension according to the procedure in CREEDS^24^. The detail is written in Supplementary Note 1 (Construction of disease-specific gene expression profiles). Gene expression data for hypertension were retrieved from GEO (GSE24752, and GSE75360 (accessed on November 9, 2022). The disease-specific gene expression levels were determined relative to a healthy cohort. Finally, we obtained the disease-specific gene expression profiles with the same gene set in CREEDS (14,804 genes) for hypertension.

### Identification of disease-specific genes from disease-specific gene expression profiles

We identified disease-specific genes for 79 diseases registered in CREEDS. Genes in CREEDS have nonzero scores indicating disease specificity. Therefore, disease-specific genes were defined as those with expression levels (not zeros) for each disease. For hypertension, we selected the top 5% of genes with positive fold change values and the top 5% of genes with negative fold change values compared to healthy control as disease-specific genes. Disease-specific genes were used to calculate network-based distance between diseases.

### Construction of disease modules using disease susceptibility genes

A set of disease susceptibility genes on the human molecular interaction network were referred to as “disease modules”^13^. To investigate the susceptibility genes of our target diseases (AML, CML, colorectal cancer, asthma, type 2 diabetes, and hypertension), we sourced relevant genes from six databases as delineated in a prior study^13^, including (i) the Online Mendelian Inheritance in Man (OMIM) database^26^, (ii) ClinVar database^27^, (iii) the genome-wide association studies (GWAS) database^28^, (iv) the phenome-wide association study database (PheWAS)^29^, (v) the GWASdb database^30^, and (vi) the DisGeNET database^31^. The accessed date and specific search keys are detailed in Supplementary Data S1. The gene symbols (HGNC symbols) were converted into Entrez IDs using the biomaRt library^32^ in R (version 4.0.3). The number of the genes in the disease module of AML, CML, colorectal cancer, asthma, type 2 diabetes, and hypertension are 1075, 51, 408, 929, 1173, and 909, respectively. A comprehensive list of genes in the modules can be found in Supplementary Data S3.

### Construction of drug response gene expression profiles

Drug-induced gene expression profiles were obtained from the LINCS Program L1000 mRNA profiling assay (http://www.lincsproject.org), where the gene expression levels for 978 landmark genes, termed L1000 genes. L1000 is highly reproducible, comparable to RNA sequencing, and suitable for computational inference of the expression levels of 81% of non-measured transcripts^33^. The gene expression profiles of L1000 were measured at various post-treatment intervals—3, 6, 24, 48, and 144 h—and across a range of concentrations using diverse human cell lines. Within the level 5 dataset, we extracted the gene expression profiles of 1488 drugs, and averaged the gene expression profiles of the same drug across experimental conditions, such as post-treatment intervals, the concentration of the drug, and cell lines. The details on drug names, efficacies, and procedures are provided in Supplementary Data S4 and Supplementary Note 1 (Construction of drug response gene expression profiles).

### Construction of drug modules using drug response genes

The top 5% of genes with positive fold change values and the top 5% of genes with negative fold change values in the drug-induced gene expression profiles were considered drug response genes and were used to construct the drug module for each drug (Supplementary Data S4).

### Evaluation of network-based distance between disease modules and drug modules

The network-based proximity between a query disease module and a drug module was evaluated using a network-based distance measure. The path length between genes constituting a disease module and drug response genes constituting a drug module was calculated. *Q* represents the set of genes ${q}_{1},\cdots ,{q}_{\left|Q\right|}$ in the query disease module, *A* represents the set of genes ${a}_{1},\cdots ,{a}_{\left|A\right|}$ in the drug A module, and $d(q,a)$ represents the shortest path length between nodes *q* and *a* in the human molecular interaction network^15^ and was defined as:

1 $$d\left(Q,\,A\right)=\frac{1}{{{{{\rm{||}}}}}A{{{{\rm{||}}}}}}{\sum}_{a\in A}{\min }_{q\in Q}d\left(q,a\right)$$

To determine the significance of the network-based proximity measure between query disease Q and drug A, a reference distance distribution was created. First, a set of genes with the same size and degree of the query disease module was randomly selected. Second, a set of genes with the same size and degree of the drug module was randomly selected. Then, the proximity between the two sets of genes in the human molecular interaction network was calculated^15^. After 100 repetitions, the mean ${\mu }_{d(Q,A)}$ and the standard deviation ${\sigma }_{d(Q,A)}$ were calculated. The normalized network-based proximity measure was defined as

2 $$z\left(Q,A\right)=\,\frac{d\left(Q,A\right)-{\mu }_{d(Q,A)}}{{\sigma }_{d(Q,A)}}$$

For a more efficient calculation of the permutation process, parallel computing using the PAR function was performed^34^. Finally, the $z\left(Q,A\right)$ sign was inverted, scaled in the range of 0 to 1, and defined as *P*_*QA*_. The code for calculation of network-based distance between disease modules and drug modules is deposited in figshare^35^.

### Evaluation of the network-based distance between diseases

The network-based proximity between query disease Q and disease *α* was evaluated using a network-based distance measure. The shortest path lengths between susceptibility genes and disease-specific genes were calculated according to formula (1). The normalized network-based proximity measure *z*(*Q*, *α*) was calculated according to formula (2). Although Sections 7 and 8 share similarities, they differ in focus. Section 7 elucidates the calculation of distance between a disease and a drug, while section 8 delves into the calculation of distance between different diseases. The code for calculation of the network-based distance between diseases is deposited in figshare^35^.

### Evaluation of network-based distance between drug modules

To evaluate the network-based distance between drug A module and drug B module, the network-based separation measure of the drug response genes between drug A module and drug B module was calculated^13^. Briefly, the network-based separation measure 〈*s*_*AB*_〉 was defined as follows:

3 $${s}_{{AB}}\equiv \left\langle {d}_{{AB}}\right\rangle -\frac{\left\langle {d}_{{AA}}\right\rangle +\left\langle {d}_{{BB}}\right\rangle }{2}$$

〈*d*_*AA*_〉 is the mean shortest distance between the response genes of drug A in a human molecular interaction network, 〈*d*_*BB*_〉 is the mean shortest distance between the response genes of drug B in a human molecular interaction network, and 〈*d*_*AB*_〉 is the mean shortest distance between the response genes of drug A and drug B.

If drug A had only a single response gene, the average shortest distance between the response genes of drug A (denoted as 〈*d*_*AA*_〉) would be 0. The computation of the distance between two drug modules was based on the response genes of drugs A and drug B. When both drugs A and drug B possessed only one response gene, and if that gene was identical, the distance between the drug modules (denoted as 〈*s*_*AB*_〉) would be 0. In this study, drug response genes were identified as the top 5% of genes exhibiting the positive or negative expression changes in the drug-induced gene expression profiles. Since the number of response genes for drug A (or drug B) ranged from 198 to 200, the associated distance was not 0.

The formulas for calculating the drug–disease distance (S-score using formula [1]) and the network-based separation measure of the drug response genes between drug A module and drug B share similarities, but they exhibit a distinction. Therefore, we used different notations. The determination of the distance between a drug module and a disease module relies on generating a random distribution of S-scores. In contrast, the distance between drugs is determined by the topological distance between one drug and another on the molecular network. This choice is motivated by the substantial number of drug pairs, where the computational expense of generating a random distribution is notably high. The code for calculation of network-based distance between drug modules is deposited in figshare^35^.

### Evaluation of the network-based disease similarity based on disease-specific gene expression profiles

The similarity between query disease module *Q* and the other disease *a* was evaluated using the network-based proximity measure *R*(*Q*, *a*)^36^. The normalized network-based proximity measure between query disease module *Q* and the other disease *a* was evaluated as *z*(*Q*, *a*). The network-based similarity was calculated by sign inversion and scaling of the proximity *z*(*Q*, *a*) in the range of 0 to 1. The similarity of 79 diseases in the CREEDS database and six diseases (AML, CML, colorectal cancer, asthma, type II diabetes, and hypertension) was calculated.

### Evaluation of drug-similarity based on the chemical structures

The structure-based similarity between drug A and drug B was evaluated as *R*(*A*,*B*) based on the chemical structures. Chemical structures (MOLfiles) for 8,287 drugs were retrieved from the KEGG DRUG^37^. KCF-S fingerprints^38^ for each drug were computed using kcfconvoy (https://github.com/KCF-Convoy/kcfconvoy). The generalized Jaccard similarity coefficient between drugs was calculated based on the fingerprints^39^.

### Evaluation of transcriptional correlations between a disease module and drug modules

The transcriptional correlation of the gene expression profiles between the query disease Q module and the drug A module was evaluated by the cosine correlation coefficient represented as *C*_*QA*_. Similarly, the transcriptional correlation between the query disease Q module and the drug B module was evaluated as *C*_*QB*_. The cosine coefficient was calculated using the cosine function in the lsa library (version 0.73.2) in R. The code for calculation of transcriptional correlations between a disease module and drug modules is deposited in figshare^35^.

### Amplification of component genes in the disease and drug modules by network propagation

The size of the query disease module and drug modules was increased by network propagation. A　network propagation method with prior knowledge, called PRINCE, was leveraged^40^. The algorithm is implemented in https://github.com/kztakemoto/network_propagation.

The network propagation with prior knowledge calculates the probability of genes belonging to the query disease module or the drug module. Neighbor nodes of a query disease module were identified as candidates for new genes of the query disease module. The network-based similarity between diseases was used as prior knowledge in the network propagation procedure. Neighbor nodes for a drug module were identified as candidates for new genes of the drug module. The structure-based similarity between drugs was used as prior knowledge for the network propagation procedure.

Network propagation without prior knowledge was also performed as follows: Neighbor nodes of a query disease module were identified as candidates for new genes of the query disease module. Similarly, neighbor nodes of a drug module were identified as candidates for new genes of the drug module. The neighbors function in the igraph library (v.1.2.6) was used to identify the neighbor nodes^41^. The parameters of network propagation are summarized in Supplementary Supplementary Data S5.

### Design of the prediction score for synergistic drug combinations

**Fig. 1:**
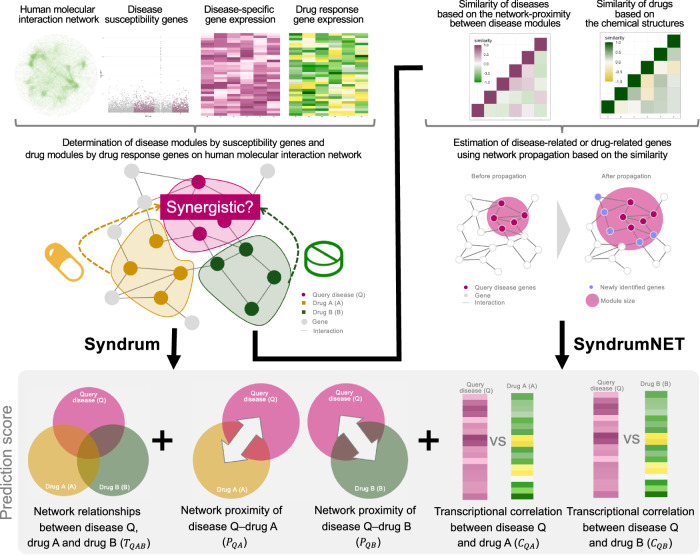
Overview of our network-based trans-omics approach to predict synergistic drug combinations. A comprehensive human molecular interaction network was constructed. Susceptibility genes and drug response genes were mapped onto the network. The network-based relationships between a query disease module and drug modules were calculated. The network-based proximities between a query disease module and drug modules were calculated. For the method without network propagation (Syndrum), the transcriptional correlations between a query disease module, and drug modules were calculated using the overlapping genes between a query disease module and the drug modules. The network-based similarities of diseases are calculated based on network-based proximity between diseases, referred to as disease similarity. The structural similarity of drugs was calculated based on the chemical structure, referred to as drug similarity. Network propagation using the similarities was performed to identify overlap genes between a query disease module and disease modules. For the method with network propagation (SyndrumNET), the transcriptional correlations between a query disease module, and drug modules were calculated using the newly identified overlapping genes. Finally, the network-based localization relationships score, the network-based proximities, and the transcriptional correlation coefficients between a query disease module and the drug modules were integrated. Q indicates ‘Query disease’, A indicates ‘Drug A’, and B indicates ‘Drug B’.

The prediction score of the synergistic effect of drug A and drug B for a query disease was designed using three components (Fig. 1). The first component was the network-based localization relationship score between a query disease module, drug A module, and drug B module, which is referred to as (*T*_*QAB*_). The second component was the network-based proximity score between a query disease module, drug A module, and drug B module, which is referred to as (*P*_*QAB*_). The third component was the transcriptional correlation coefficient between a query disease module, drug A module, and drug B module (*C*_*QAB*_).

Network-based localization relationship score (*T*_*QAB*_) was determined based on the topological classes of the query disease module, drug A module, and drug B module. Six types of topological classes (Class I ~ Class VI) were defined based on a previous study^13^. Within the six classes, class II is designated as Complementary Exposure and it tends to have synergistic effects. This class represents situations where two separated drug modules that overlap individually with a query disease module ($\normalsize z\left(Q,A\right) \, < \, 0, z \left(Q,B\right) \, < \, 0 \, {and} \; {s}_{{AB}} \, < \, 0$). Based on the topological classes, the score of the network-based localization relationship was assigned as follows:

4 $${T}_{{QAB}} = \left\{\begin{array}{ll}0 \hfill & \left( {when}\, {other}\, {class}\right) \\ 2 & \left({when} \, {class}\, {II}\right)\hfill\end{array}\right.$$

The network-based proximity score (*P*_*QAB*_) was calculated by averaging the network-based proximity between the query disease Q module and drug A module (*P*_*QA*_) and the network-based proximity between the query disease Q module and drug B module (*P*_*QB*_), as follows:

5 $${P}_{{QAB}}=\frac{\left({P}_{{QA}}\,+{P}_{{QB}}\right)}{2}$$

The transcriptional correlation score (*C*_*QAB*_) was calculated by averaging the absolute value of the transcriptional correlation coefficient between the query disease Q module and drug A module (*C*_*QA*_) and the transcriptional correlation coefficient between the query disease Q module and drug B module (*C*_*QB*_), as follows:

6 $${C}_{{QAB}}=\frac{\left(\left|{C}_{{QA}}\,\right|+\left|{C}_{{QB}}\right|\right)}{2}$$

Finally, the prediction score was calculated by adding the network-based localization relationship score (*T*_*QAB*_), network-based proximity score (*P*_*QAB*_), and transcriptional correlation score (*C*_*QAB*_) as follows^35^:

7 $${Predction\; score}=\,{{T}_{{QAB}}+{P}_{{QAB}}+C}_{{QAB}}$$

### Collection of drug combinations with known synergistic effects for various diseases

The known synergistic drug pairs for AML, CML, colorectal cancer, asthma type 2 diabetes, and hypertension were obtained from the PubMed database. The keywords “synergy,” “synergic,” “synergistic,” “synergism,” “interaction,” and “combination” along with disease names were used as keywords for the search procedure. For AML and CML, known synergistic drug pairs were retrieved from DrugCombDB (2019.05.31 release version)^42^. DrugCombDB contains drug combinations for human cancer cell lines. We linked the drugs and diseases according to the cell line. For hypertension, known synergistic drug pairs were retrieved from a previous paper^13^. The curated known synergistic drug pairs are summarized in Supplementary Data S6.

### Performance evaluation protocol

The area under the receiver operating characteristic curve (AUC) was calculated using the performance function in the ROCR library (v.1.0-11) in R. The code for the performance evaluation is deposited in figshare^35^.

### Chemicals used for the cell survival assay

Capsaicin was purchased from FUJIFILM Wako Pure Chemical Industries, Ltd. (Osaka, Japan). Daunorubicin hydrochloride, idarubicin, and topotecan were purchased from Cayman Chemicals (Ann Arbor, Michigan, USA). Mitoxantrone, fasudil were purchased from Tokyo Chemical Industries Co., Ltd. (Tokyo, Japan). Cell Counting Kit-8 used for the WST-8 assay was purchased from DOJINDO (Tokyo, Japan).

### Cell culture and reagents

The K562 human CML cell line was obtained from the RIKEN BioResource Center (Tokyo, Japan) and grown in RPMI 1640 (NACALAI TESQUE, INC., JAPAN) medium supplemented with 10% fetal bovine serum (Funakoshi Co., Ltd., JAPAN). All cells were incubated at 37 °C in a humidified atmosphere containing 5% (v/v) CO_2_.

### Cell survival assay

In vitro growth inhibition was evaluated according to the manufacturer’s standard protocol using the Cell Counting Kit-8. Cells were seeded in 96-well plates at a density of 5000 cells/well in a total volume of 100 µL and exposed to various drugs for 72 h at 37 °C in a 5% CO_2_ atmosphere. WST-8 was added, and after 3 h, the absorbance was measured at a wavelength of 450 nm (reference 630 nm) using a microplate reader (Bio-Rad Laboratories, Inc., Hercules, CA). The results are expressed as percentages [i.e., as the ratio of the absorbance of treated cells to that of the control (drug untreated group, 100%)]. Percent survival was calculated using the following formula: percentage survival = (absorbance of treated wells − absorbance of blank wells)/(absorbance of untreated wells − absorbance of blank wells) × 100. The number of biological replicates is three.

### Statistical evaluations of the significance of combinatory effects for drug synergy

Two essential models were used to evaluate the significance of combinatory effects for drug synergism: Bliss’s IA^43^ model and Loewer’s additivity (CA) model^44^.

For Bliss’s IA model, it is assumed that the effects of drugs are stochastic events under the non-interaction assumption between drugs. The effects of a drug combination are calculated as the joint probability of each effect. Drug A causes v% effects and drug B causes w% effects at a given combination of concentrations. The total effect rate of the combination can be computed as ${{CI}}_{{mix}}=1-\left(1-v\right)\left(1-w\right)$ under the additive assumption. Thus, if ${{CI}}_{{mix}} \, > \, 1$, a given drug combination is considered to have synergistic effect based on Bliss’s IA model. ${{CI}}_{{mix}}$ is denoted as the IA score in this study.

For Loewer’s additivity model, it is assumed that the toxic unit equals one under the non-interaction assumption between drugs. The concentrations weighted by the effect of each drug (toxic unit [TU]) are added together to yield the TU of a given drug combination.

$${{{\mbox{Toxic}}}} {{{{\rm{Unit}}}}} {({{\mbox{TU}}})} = \frac{{C}_{a}}{{{EC}}_{u}^{A}}+\frac{{C}_{b}}{{{EC}}_{u}^{B}}$$

For a given drug combination, *C*_a_ and *C*_b_ stand for the concentrations of drug A and drug B, respectively. ${{EC}}_{u}^{A}$ and ${{EC}}_{u}^{B}$ are the concentrations of the drugs causing u% effect by drug A and drug B, respectively. If TU = 1, the effect rate of the drug combination remains at u% under the additive assumption. Thus, if TU < 1, a given drug combination is assumed to have synergistic effect based on Loewer’s additivity (CA) model, when u% is selected as the results of the growth inhibition assay. ${{\mbox{TU}}}$ is denoted as the CA score in this study.

### Sample preparation for microarray analysis

Cells were seeded at a density of 50,000 cells/mL in a 10-cm diameter dish and exposed to various drugs (capsaicin 50 μM, mitoxantrone 30 nM) for 24 h at 37 °C in a 5% (v/v) CO_2_ atmosphere. Experiments were conducted in three independent wells for each group. Total RNA was extracted using the RNeasy Mini Kit (QIAGEN, Valencia, CA) according to the manufacturer’s protocol and used for microarray experiments by the well. The extracted total RNA from these wells was combined by exposure condition.

### Mode-of-action of drug combinations by microarray analysis

Cyanine-3 (Cy3) labeled cRNA was prepared from 150 ng RNA using the One-Color Low Input Quick Amp Labeling kit (Agilent) according to the manufacturer’s instructions, followed by RNAeasy column purification (QIAGEN, Valencia, CA). Dye incorporation and cRNA yield were assessed using a NanoDrop ND-1000 Spectrophotometer.

Cy3-labeled cRNA (600 ng, specific activity >6 pmol Cy3/µg cRNA) was fragmented at 60 °C for 30 min in a reaction volume of 25 µl containing 25× Agilent fragmentation buffer and 10× Agilent blocking agent following the manufacturer’s instructions. Upon completion of the fragmentation reaction, 25 µl of 2× Agilent hybridization buffer was added to the fragmentation mixture and hybridized to Agilent SurePrint GE Unrestricted Microarrays (G2519F) for 17 h at 65 °C in a rotating Agilent hybridization oven. After hybridization, microarrays were washed for 1 min at room temperature with GE Wash Buffer 1 (Agilent) and 1 min at 37 °C with GE Wash Buffer 2 (Agilent), then dried immediately.

The slides were scanned immediately after washing using an Agilent DNA Microarray Scanner (G2505C) with a one-color scan setting for 8 × 60 K array slides (Scan Area 61 × 21.6 mm, Scan resolution 3 µm, Dye channel was set to Green and Green PMT was set to 100%).

The scanned images were analyzed with Feature Extraction Software 10.7.1.1 (Agilent) using default parameters (protocol GE1_107_Sep09 and Grid: 028282_D_F_20110531) to obtain background subtracted and spatially detrended processed signal intensities. Features flagged in Feature Extraction as Feature Non-uniform outliers were excluded. Our microarray data have been registered and are publicly available in the Gene Expression Omnibus (GEO) database (GSE254052). We calculated the log2 fold change (log_2_FC) for each probe when comparing the control and exposed groups. Then, we averaged the log_2_FC for genes. We defined differential expression genes (DEGs) as genes with absolute value of log_2_FC greater than one ($\left|{\log }_{2}{FC}\right|\ge \,1$) in the exposed group compared to the control group.

### Enrichment analysis of transcription factors

Gene set enrichment analysis was performed using DAVID^45^ and “clusterProfiler” (v4.4.4) library in R^46^. The transcription factor enrichment analysis was performed using ChEA^47^. The results were considered statistically significant at *p* < 0.05.

### Statistics and reproducibility

We performed two-sided t-test using “stats” (v4.3.1) library in R and Fisher’s exact test using DAVID^45^ and “clusterProfiler” (v4.4.4) library in R^46^. We examined whether the mean distance from a disease to a drug with a known effect differs from that to a drug with an unknown effect by two-sided t-test. For t-test, the sample size is 1,106,328 drug pairs for each query disease. For Fisher’s exact test using DAVID^45^ and “clusterProfiler”, the sample size is the number of genes of functional pathways in the KEGG database, totaling 8,156 and 8,772 genes, respectively. We conducted cell survivable assay following microarray analysis. For cell survivable assay, we preformed three biological replicates. The mRNA from these replicates were mixed and used for microarray analysis.

### Inclusion & ethics statement

All members involved in this study have met the authorship criteria mandated by Nature Portfolio journals and have been listed as authors. Their contributions were vital to the study’s design and execution. The roles and responsibilities of each collaborator were clearly defined and mutually agreed upon before initiating the research. This research faced no severe restrictions or prohibitions within our operational environment and was conducted in a manner that avoids causing stigmatization, incrimination, discrimination, or personal risk to any parties involved. In preparing our manuscript, we diligently referenced research that aligns with our study, ensuring that our citations reflect the pertinent scientific context and contributions.

## Results

### Overview of the proposed trans-omics methods

We propose “SyndrumNET”, a network-based trans-omics approach, to predict synergistic drug combinations by integrating genome, transcriptome, interactome, and diseasome. An overview of the proposed method is shown in Fig. 1, and the detailed procedures are described in the Methods section. Disease susceptibility genes and drug target genes are not randomly dispersed throughout the human molecular interaction network. Instead, they form localized clusters, termed either disease modules or drug modules^13,48^. If two drug modules are close to a disease module but distant from each other, these two drugs tend to have synergistic effects on the disease^13^.

In the first step, we aimed to understand the relationships between a disease and drugs based on their localization in the human molecular interaction network. We constructed a comprehensive human molecular interaction network by integrating various types of molecular interactions (e.g., physiological protein-protein interactions) from multiple databases (Fig. 1, Supplementary Data S1, Supplementary Data S2 and see Methods). Then, we identified disease modules and drug modules using disease susceptibility genes and drug response genes (Supplementary Data S3, Supplementary Data S4 and see Methods). We measured the network-based proximity between a query disease module and drug modules and network-based separation between drug modules. We evaluated the relationships between a disease and drugs based on their localization in the network, which we term as the network-based localization relationships between a query disease module and the drug modules.

In the second step, we aimed to understand the relationships between a disease and drugs based on their proximity in the human molecular interaction network. The network-based drug-disease proximity can quantify the influence of the drug on the disease^15^. We averaged the network-based proximity between a query disease and each of the two drug modules (Fig. 1 and Methods).

In the third step, we evaluated the transcriptional correlations between a query disease module and the drug modules. The gene expression profiles for the diseases were constructed from Crowd Extracted Expression of Differential Signatures (CREEDS)^24^ and the gene expression profiles for the drugs were constructed from the Library of Integrated Network-Based Cellular Signatures (LINCS)^49^. There is a limitation to the number of genes that overlap between a query disease module and the drug modules. To overcome this problem, we amplified the number of overlapping genes between a query disease module and the drug modules by network propagation with the similarities of diseases and drugs (Fig. 1 and Methods).

Finally, we developed a scoring scheme by integrating the network-based proximity and the transcriptional correlations between a query disease module and the drug modules (see Methods). The proposed method without network propagation is referred to as Syndrum, while the proposed method with network propagation is referred to as SyndrumNET. We applied these methods to predicting synergistic drug combinations for six diseases including AML, CML, colorectal cancer, asthma, type 2 diabetes, and hypertension.

### Synergistic drug combinations can be explained by the topological relationship between drug modules and disease modules in the human molecular interaction network

We examined the relationships between disease modules and drug modules for six diseases and 1488 drugs, including AML, CML, colorectal cancer, asthma, type 2 diabetes, and hypertension. We calculated the network-based proximity between a query disease module and its approved drug modules using the shortest path length (see Methods). Then, we compared the results between approved drugs and the other drugs with respect to the network-based proximity for a query disease. For cancers and asthma, a query disease and its approved drugs are closely located compared with the other drugs in terms of network-based proximity (Fig. S1). The results suggest that drug response genes tend to be close to susceptibility genes for cancers and asthma if the drugs are effective for the treatment of the disease.

**Fig. 2:**
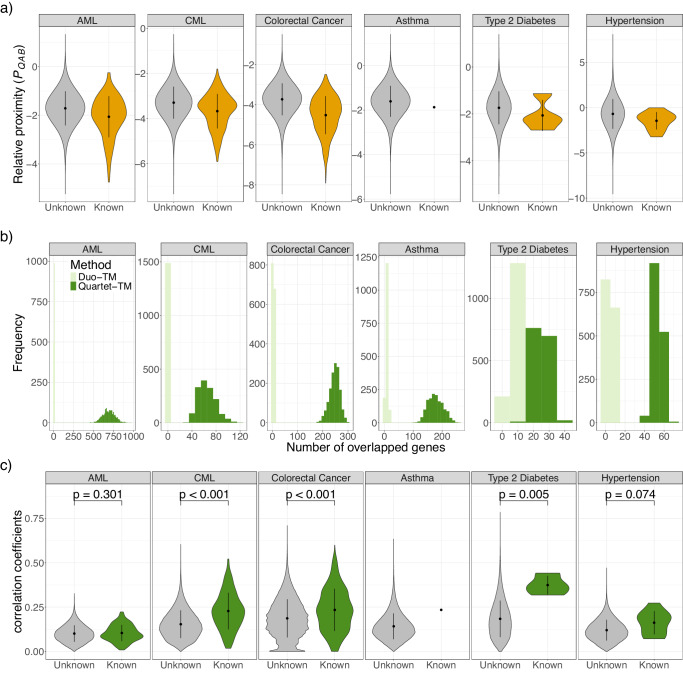
Distributions of average network-based proximity (*P*_*QAB*_), the number of overlapped genes and absolute transcriptional correlation coefficients between a query disease module (Q) and drug modules (A and B). **a** Distribution of average network-based proximity (*P*_*QAB*_) between a query disease module (Q) and drug modules (A and B). Gray color indicates the network-based proximity between a query disease module and drug modules with unknown effects (AML: 1,106,100 pairs, CML: 1,106,103 pairs, colorectal cancer: 1,105,598 pairs, asthma: 1,106,327 pairs, type 2 Diabetes: 1,106,324 pairs, hypertension: 1,106,318 pairs). The orange color indicates the network-based proximity between a query disease module and drug modules with known synergistic effects (AML: 228 pairs, CML: 225 pairs, colorectal cancer: 730 pairs, asthma: 1 pair, type 2 Diabetes: 4 pairs, hypertension: 10 pairs). Upper and lower whiskers mean standard deviations, and points indicate mean. **b** Distribution of the number of overlapped genes between a query disease module (Q) and individual drug modules (A or B). Light green represents the number of overlapped genes in the method without network propagation (Syndrum). Green represents the number of overlapped genes in the method with network propagation (SyndrumNET). **c** Distribution of average absolute transcriptional correlation coefficients between a query disease module (Q) and drug modules (A and B). Gray color indicates the average absolute transcriptional correlation coefficients between a query disease module and drug modules with unknown synergistic effects. Green color indicates the average absolute transcriptional correlation coefficients between a query disease module and drug modules with known synergistic effects. The number of pairs of each group is consistent with Fig. 2a. Upper and lower whiskers mean standard deviations, and points indicate mean. The *p*-values were calculated using two-sided t-test.

Next, we examined the network-based proximity relationships between 1488 drug modules and six disease modules with respect to known drug synergy. For a query disease, we compared the averaged network-based proximity between drug pairs with known synergistic effects and the other drug pairs. This tends to be shorter compared with that of randomly selected drug module pairs (Fig. 2a). The results suggest that synergistic drug combinations can be explained by the distance between a disease module of interest and drug modules in the human molecular interaction network.

### Network propagation with disease similarities and drug similarities emphasizes the transcriptional correlation between a query disease module and drug modules

We examined the transcriptional correlations between each query disease module and individual drug modules using the overlapped genes in the gene expression profiles. We identified overlapping genes between 1,488 drug modules and each of the six diseases. There are less than ten overlapped genes for each query disease module with the drug modules for most disease–drug pairs (Fig. 2b and Fig. S2a).

We obtained the averaged transcriptional correlation coefficients for 1,106,328 drug pairs for the query disease and compared these coefficients between known synergistic drug pairs and the other drug pairs. We observed no significant difference between drug pairs with synergistic effects and those without synergistic effects (Fig. S2b). This observation may be due to the low number of overlapping genes between the query disease and drug modules.

We amplified the number of overlapping genes between the query disease module and drug modules by network propagation. We performed network propagation with prior knowledge on disease similarities and drug similarities (see Methods), which determines the prioritization of genes belonging to the query disease module or the drug module^40^. Based on the rank, we identified neighbor nodes for a query disease module as candidates for new genes. A summary of the parameters of network propagation are listed in Supplementary Data S5. The number of overlapped genes between the query module and drug modules is greater than ten for all disease–drug pairs (Fig. 2b and Fig. S2c). The network propagation process successfully increased the number of overlapped genes between the query module and drug modules.

Next, we examined the transcriptional correlation between each query disease and the individual drug modules using newly identified genes in the modules (Fig. 2c and Fig. S2d). We then averaged the transcriptional correlation coefficients of two drug modules for the query disease and obtained the averaged transcriptional correlation coefficients for 1,106,328 drug pairs for the query disease. We observed that the averaged transcriptional correlation coefficients of drug pairs with synergistic effects are significantly higher than those without synergistic effects (Fig. 2c) (*p*-value for CML < 0.001, colorectal cancer <0.001, and type 2 diabetes = 0.005 by two-sided t-test), except for AML and hypertension (*p*-value for AML = 0.301 and hypertension = 0.074 by two-sided t-test). These results suggest that network propagation emphasizes the strength of transcriptional correlations between a disease and drugs in the human molecular interaction network.

### Performance evaluation of the proposed network-based trans-omics approach

We evaluated the performance of our proposed network-based trans-omics methods, Syndrum and SyndrumNET, to identify synergistic drug combinations among 1,488 drugs (Table 1). We focused on six diseases with at least one known synergistic drug combinations (Supplementary Data S6). We compared the prediction performance between the previous method, Syndrum, and SyndrumNET. Note that the previous method corresponds to the use of the network-based separation between drug modules^13^.

**Table 1:** Comparison of the AUC between the proposed method and previous methods

Method	Omics data	Considered relationships	AML (228)	CML (225)	Colorectal cancer (735)	Asthma (1)	Type 2 Diabetes (4)	Hyper-tension (10)
Previous (Cheng et al.)^13^	Interactome		0.471	0.499	0.487	0.689	0.340	0.628
Syndrum	Interactome + transcriptome		0.519	0.559	0.686	0.542	0.839	0.507
SyndrumNET	Interactome + transcriptome + diseasome		**0.605**	**0.741**	**0.777**	**0.870**	**0.929**	**0.715**

Brackets shows the number of drug combinations with known synergistic effects.

Bold shows the method has the best AUC in each disease.

demonstrates network-based relationships between disease and drugs

demonstrates network-based separation between drugs.

demonstrates network-based proximity of disease and drugs.

indicates network-based proximity of disease and drugs

indicates network propagation.

SyndrumNET works the best (Table 1). The prediction accuracy increased by 31.4% on average compared with the previous method. In particular, the AUC score for type 2 diabetes is increased 63.4% by SyndrumNET compared with the previous method (Table 1). These results suggest that it is important to consider various biological processes represented by multi-omics data for predicting synergistic drug combinations.

SyndrumNET is superior to Syndrum, which suggests that the enhancement of overlapped genes between the disease module and drug modules contributes to the enhancement of prediction accuracy (Table 1). The use of disease and drug similarities in the network propagation is more useful for predicting synergistic drug combinations.

### Comprehensive prediction of new drug combinations for six diseases

Using SyndrumNET, we predicted new synergistic drug combinations for six diseases (Supplementary Data S7). For AML, the top 20 drug pairs with antineoplastic and antihypertensive activity are predicted (Supplementary Data S7). For CML, analgesic, antibiotic, antineoplastic, and vasodilator drugs are among the top 20 (Table 2 and Supplementary Data S7). For colorectal cancer, most of the top 20 drug pairs are known antineoplastic medications (Supplementary Data S7). For asthma, anti-inflammatory drugs are among the predicted top 20 pairs (Supplementary Data S7). For type 2 diabetes, combinations of antihyperlipidemic drugs and a vitamin E supplement, tocopherol, are among the top 20 predicted drugs. In particular, the combination of an antihyperlipidemic drug (gemfibrozil) and supplement (tocopherol), which are commonly used for the treatment of diabetes separately^50^, are among the top five (Supplementary Data S7). For hypertension, many antineoplastic drug combinations are predicted in the top 20. An antihypertensive drug, zofenopril, and an antineoplastic drug is predicted as the fifth combination (Supplementary Data S7).

**Table 2:** Predicted drug combinations for CML and the experimental validation results

Rank	Drug A	Drug B	Efficacy of Drug A	Efficacy of Drug B	In silico prediction						In vitro validation				
					P_QA_^a^	P_QB_^b^	S_AB_^c^	C_QA_^d^	C_QB_^e^	Prediction score	Survival ratio of Drug A	Survival ratio of Drug B	Survival ratio of Combination	CA score^f^	IA score^g^
1	Capsaicin	Mitoxantrone	Analgesic	Antineoplastic	−7.72	−5.56	0.03	−0.18	−0.47	3.18	50.9 ± 1.3	51.9 ± 2.4	12.6 ± 2.3	**0.46**	**13.8**
2	Idarubicin	Mitoxantrone	Antibiotic	Antineoplastic	−5.13	−5.56	0.02	−0.46	−0.47	3.15	81.1 ± 2.3	54.8 ± 3.2	39.5 ± 2.4	1	**4.9**
3	Capsaicin	Idarubicin	Analgesic	Antibiotic	−7.72	−5.13	0.03	−0.18	−0.46	3.15	50.0 ± 2.1	65.1 ± 4.2	21.5 ± 2.2	**0.83**	**11.1**
4	Daunorubicin	Mitoxantrone	Antibiotic	Antineoplastic	−4.79	−5.56	0.01	−0.48	−0.47	3.13	69.6 ± 2.2	54.5 ± 2.7	35.9 ± 2.9	**0.55**	**2.0**
5	Mitoxantrone	Topotecan	Antineoplastic	Antineoplastic	−5.56	−6.23	0.02	−0.47	−0.29	3.13	61.1 ± 4.1	59.4 ± 1.9	40.5 ± 3.4	**0.81**	−4.2
6	Capsaicin	Daunorubicin	Analgesic	Antibiotic	−7.72	−4.79	0.03	−0.18	−0.48	3.13	51.2 ± 2.2	85.0 ± 5.2	20.7 ± 2.3	**0.73**	**22.8**
7	Capsaicin	Topotecan	Analgesic	Antineoplastic	−7.72	−6.23	0.03	−0.18	−0.29	3.13	55.8 ± 0.9	61.0 ± 4.3	28.1 ± 2.5	**0.48**	**5.9**
8	Fasudil	Mitoxantrone	Vasodilator	Antineoplastic	−4.16	−5.56	0.04	−0.50	−0.47	3.10	39.5 ± 2.3	60.0 ± 3.0	22.7 ± 2.3	**0.69**	**1.0**
9	Capsaicin	Fasudil	Analgesic	Vasodilator	−7.72	−4.16	0.04	−0.18	−0.50	3.10	53.8 ± 3.3	39.5 ± 2.3	15.0 ± 1.0	1.02	**6.3**
10	Daunorubicin	Idarubicin	Antibiotic	Antibiotic	−4.79	−5.13	0.01	−0.48	−0.46	3.10	81.7 ± 6.0	67.8 ± 3.2	20.5 ± 2.9	**0.37**	**34.9**
11	Idarubicin	Topotecan	Antibiotic	Antineoplastic	−5.13	−6.23	0.02	−0.46	−0.29	3.10	65.9 ± 3.5	59.4 ± 1.9	42.7 ± 4.9	**0.77**	−3.6
12	Mitoxantrone	Zofenopril	Antineoplastic	Antihypertensive	−5.56	−6.70	0.02	−0.47	0.16	3.10	64.6 ± 4.3	109.2 ± 2.3	54.3 ± 5.9	1.72	**16.1**
13	Acemetacin	Mitoxantrone	Analgesic	Antineoplastic	−5.12	−5.56	0.04	−0.37	−0.47	3.10	92.3 ± 5.5	64.6 ± 4.3	47.7 ± 3.6	1.15	**11.9**
14	Capsaicin	Zofenopril	Analgesic	Antihypertensive	−7.72	−6.70	0.02	−0.18	0.16	3.10	54.0 ± 1.5	84.1 ± 3.6	29.2 ± 5.3	**0.91**	**16.2**
15	Capsaicin	Acemetacin	Analgesic	Analgesic	−7.72	−5.12	0.03	−0.18	−0.37	3.10	54.0 ± 1.5	52.4 ± 3.0	19.3 ± 2.4	**0.67**	**9.0**
16	Fludarabine	Mitoxantrone	Antineoplastic	Antineoplastic	−6.97	−5.56	0.03	−0.12	−0.47	3.10	22.2 ± 1.0	55.4 ± 4.0	4.3 ± 1.2	**0.33**	**8.0**
17	Mitoxantrone	Tiludronic acid	Antineoplastic	Antiresorptive	−5.56	−5.41	0.04	−0.47	0.32	3.10	—	—	—	—	—
18	Capsaicin	Fludarabine	Analgesic	Antineoplastic	−7.72	−6.97	0.03	−0.18	−0.12	3.10	58.6 ± 2.0	66.0 ± 3.3	17.0 ± 2.3	**0.8**	**21.7**
19	Capsaicin	Tiludronic acid	Analgesic	Antiresorptive	−7.72	−5.41	0.04	−0.18	0.32	3.10	—	—	—	—	—
20	Mitoxantrone	Propranolol	Antineoplastic	Antiarrhythmic	−5.56	−4.37	0.03	−0.47	0.46	3.09	—	—	—	—	—

^a^P_QA_: network-based proximity between the query disease Q module and drug A module.

^b^P_QB_: network-based proximity between the query disease Q module and drug B module.

^c^S_AB_: network-based separation measure between drug A and drug B module.

^d^C_QA_: transcriptional correlation coefficient between the query disease Q module and drug A module.

^e^C_QB_: transcriptional correlation coefficient between the query disease Q module and drug B module.

^f^CA score: additivity model score.

^g^IA score: independent action score.

Bold shows the combination has synergistic efficacy.

We examined the prediction results for CML in more detail. The combination of capsaicin and mitoxantrone is predicted to be the top-ranked pair. Mitoxantrone is an antitumor drug used for AML. Furthermore, the combination of topotecan and mitoxantrone is ranked fifth. A preclinical study of CML patients revealed that this combination exhibited modest activity in the accelerated phase of CML^51^. This suggests that SyndrumNET successfully reproduced a known synergistic drug combination with a high score.

### Drug combinations may exert synergistic effects by targeting specific regions of functional pathways

**Fig. 3:**
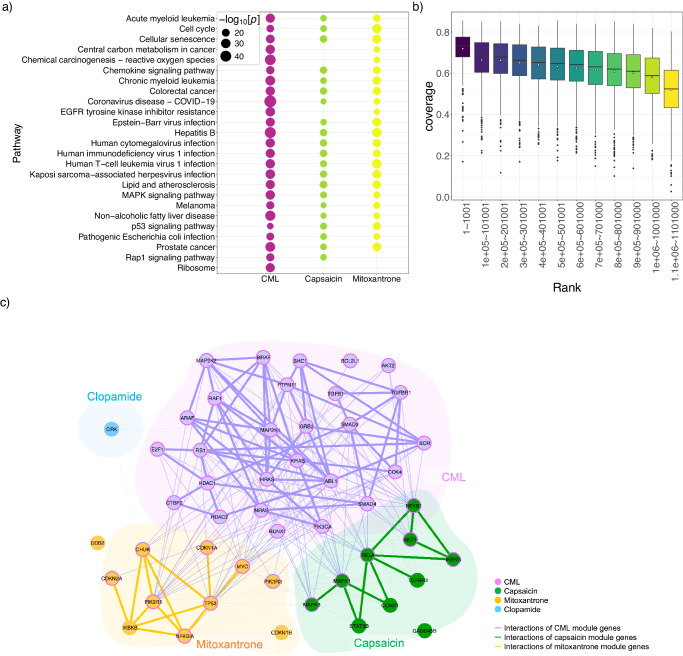
The functional pathway and relationships of CML module and drug modules in the human interactome. **a** Enriched functional pathways in the CML module, and the capsaicin and mitoxantrone modules in the method with network propagation (SyndrumNET). Purple color indicates CML. Light green color indicates capsaicin. Yellow color indicates mitoxantrone. The *p*-value calculated by Fisher exact test using clusterProfiler^46^. The sample number is 8,772 genes. The size of points reflects the *p*-value of the enrichment analysis. **b** Distribution of the coverage of enriched pathways between the CML module and drug modules. Vertical axis indicates coverage of the functional pathways by drug module pairs. Horizontal axis indicates rank of the drug module pairs when sorted in order of the coverage. Center line, median; box limits, upper and lower quartiles; whiskers, 1.5× interquartile range; white points, mean; black points, outliers. **c** Network-based relationship between the CML module and a synergistic drug combination, capsaicin and mitoxantrone, in the chronic myeloid leukemia pathway (hsa05220). Purple-colored nodes indicate CML module genes. Green-colored nodes indicate capsaicin module genes and orange-colored nodes represent mitoxantrone module genes. Aqua-colored nodes indicate clopamide module genes. The purple edged nodes indicate that the gene belongs to the CML module. The purple color edges show the interaction between the genes in the CML module. Green and orange color edges represent the interaction between the module genes of capsaicin and mitoxantrone, respectively.

We examined the relationships between the query disease module and two drug modules with respect to functional pathways in the case of CML, for example. First, we performed a functional pathway enrichment analysis of the genes associated with the CML, capsaicin, and mitoxantrone modules. Using the Syndrum method, 35 pathways including the leukemia-related pathway and the Ras1 signaling pathway are enriched in the CML module (Fig. S3 and Supplementary Data S7). Eleven pathways, such as the p53 signaling pathway, are enriched in the capsaicin module (Fig. S3 and Supplementary Data S7). Fifty-nine pathways, including human T-cell leukemia virus 1 infection, are enriched in the mitoxantrone module (Fig. S3 and Supplementary Data S7). Using the SyndrumNET method, the number of pathways identified in the CML, capsaicin, and mitoxantrone modules are 187, 138, and 145, respectively (Fig. 3a, Fig. S3 and Supplementary Data S7). These results suggest that network propagation increased the diversity of functional pathways enriched in the modules (Fig. 3a).

Second, we examined the relationship of the functional pathways between the CML disease module and two drug modules by calculating the coverage of the enriched functional pathways (Fig. 3b). For example, 169 functional pathways are enriched in the capsaicin and mitoxantrone modules, of which 151 pathways are also enriched in the CML module. The functional pathway coverage of the CML module by capsaicin and mitoxantrone modules was 0.81. The functional pathway coverage was decreased along with the predicted rank (Fig. 3b). This suggests that the top predicted drug pairs tend to target functional pathways enriched in the CML module.

Next, we determined whether the genes in the query disease and drug modules were clustered into specific regions of a functional pathway. We measured the size and significance of the largest connected component (LCC) formed by the genes of the disease module and two drug modules in a functional pathway. The module genes of CML, capsaicin, and mitoxantrone formes a larger LCC than expected by chance (Z-score > 1.95) in 50 of the 187 CML-enriched pathways. For example, the genes associated with the capsaicin and mitoxantrone modules are in the neighborhood of genes in the CML module and are clustered in the chronic myeloid leukemia pathway (hsa05220) (Fig. 3c). On the other hand, the genes in the clopamide module are located aside from the CML module in the functional pathway (Fig. 3c). These results suggest that the genes in the capsaicin and mitoxantrone modules are specifically localized into the neighborhoods of the functional pathways enriched in the CML module.

### In vitro experimental validation of the predicted drug combinations for CML

We validated the prediction results of SyndrumNET in vitro by conducting cell survival assays on CML cells (K562) for the top 20 predicted drug combinations (Table 2). Tiludronic acid was excluded from the validation list because it is not considered to act directly on cancer cells as it exhibits a preferential effect on skeletal muscle cells. Propranolol was also excluded from the validation list because it lowers blood pressure by blocking beta-adrenergic receptors in the heart and suppressing the heartbeat. In addition, cancer patients often experience cardiac dysfunction resulting from anticancer drug exposure and other pathological conditions, such as cancer cachexia. Among the top 20 predicted drug combinations, three pairs include either tiludronic acid or propranolol. The number of excluded combinations was 3, leaving 17 combinations for the final validation.

We exposed K562 cells to the drug pairs above for 72 h and measured cell survival using the WST assay. The survival ratio for each drug on K562 cells is shown in Table 2 as a ratio of drugs A or B. In addition, cell viability for the drug combination is shown as a survival ratio (Table 2). The statistical significance of synergistic effects for the top 17 drug combinations were evaluated using the CA model^44,52^ and the independent action (IA) model^43,52^, which are standard indicators of drug synergy. The output from the CA model is referred to as a CA score. If the CA score is lower than 1, the corresponding drug pair is considered to have a synergistic effect. The smaller the CA score, the higher the synergy. The output from the IA model is referred to as the IA score. If the IA score is higher than 1, the corresponding drug pair is considered to exhibit synergy. Synergistic effects were observed for 76.5% of the drug combinations in the CA model and 88.2% in the IA model. These results demonstrate that our prediction approach has high accuracy.

### Elucidation of the mode-of-action of the synergistic drug combination for CML by microarray analysis

**Fig. 4:**
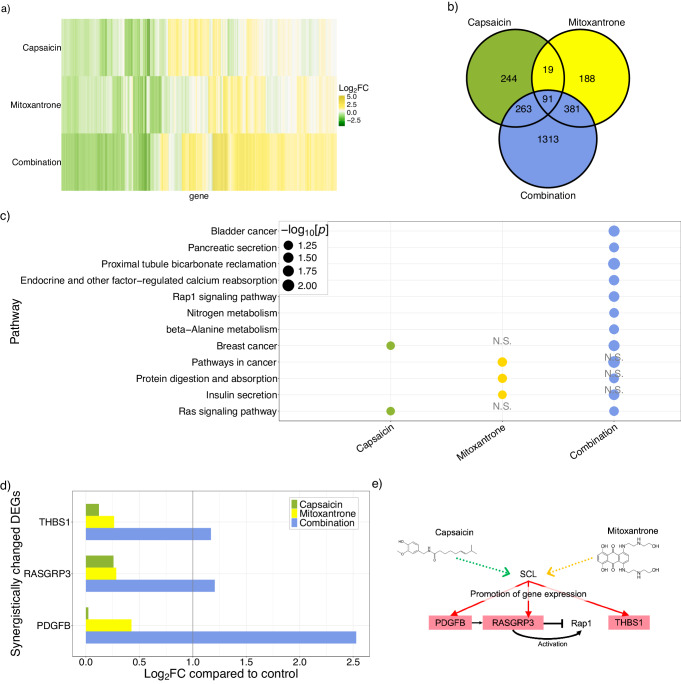
Microarray-based mode-of-action analyses. **a** The heatmap of log_2_FC of capsaicin, mitoxantrone, and the combination exposure group. Yellow and green colors indicate up- and down-regulation, respectively. **b** The number of DEGs and overlapping genes between the three exposure groups. Cyan represents the combination exposure group. Green represents the capsaicin exposure group. Yellow represents the mitoxantrone exposure group. **c** The significance of the pathways enriched only in the combination exposure group. Cyan represents the combination exposure group. Green represents the capsaicin exposure group. Yellow represents the mitoxantrone exposure group. N.S. indicates not significant based on the Fisher exact test (p > 0.05) using DAVID^45^. The number of samples is 8,156 genes. **d** The log_2_FC of *THBS1, RASGRP3*, and *PDGFB* that belong to the RAP1 singling pathway and thought to be synergistically regulated by the combination exposure group. Green, yellow, and cyan represent capsaicin, mitoxantrone, and the combination exposure groups, respectively. Exposure experiments were conducted in three independent wells for each exposure condition. The extracted total RNA from these wells was combined by exposure condition for microarray analysis. Thus, the sample number for this figure is one. **e** The mode-of-action of the effects of combination exposure. The red solid lines represent the connections predicted by the transcription factor enrichment assay. The black solid lines indicate the known interactions between the proteins. The green- and orange-colored lines represent the assumed connection between the transcription factor and capsaicin and mitoxantrone, respectively.

We examined the mode-of-action of synergistic drug combinations by microarray analysis. We focused on the drug combination of capsaicin and mitoxantrone, which were the top predicted drug pair identified by SyndrumNET. To determine the transcriptomic responses of CML cells to capsaicin, mitoxantrone, and the combination, we conducted a microarray analysis of CML cells exposed to the individual drugs and the combination. We identified 617 differentially expressed genes (DEGs) for capsaicin (Fig. 4a, b and Supplementary Data S8), 679 for mitoxantrone (Fig. 4a, b, and Supplementary Data S8), and 2,048 associated with the combination (Fig. 4a, b, and Supplementary Data S8). Moreover, 91 DEGs are common to the three groups (Fig. 4b), whereas 1,313 DEGs are detected only in the combined exposure group (Fig. 4b). The DEGs in the combined exposure group are different from those in the capsaicin and mitoxantrone exposure groups. These results suggest that the mechanism underlying transcriptional changes may be different between single and combined drug exposure.

Next, we investigated the functional pathways that were synergistically regulated by exposure to the drug combination (Table 3). Functional pathways enriched in the combined exposure group, but not in the single exposure group, are considered synergistically regulated. We identified 12 functional pathways that are synergistically affected in the combined exposure group. Interestingly, the Rap1 signaling pathway is identified only in the combined exposure group, but not in the single exposure groups (Table 3 and Fig. 4c). The Rap1 signaling pathway plays an essential role in the migration of leukocytes and lymphocytes and in the regulation of tumor progression^53–55^. These results suggest that the 12 functional pathways including the Rap1 signaling pathway are important to the synergistic effects of the drug combination in CML.

**Table 3:** The enriched functional pathways in the DEGs

class	Capsaicin	Mitoxantrone	Combination	Pathways
I	✓	✓	✓	hsa05202:Transcriptional misregulation in cancer
II	✓		✓	hsa04060:Cytokine-cytokine receptor interaction, hsa04061:Viral protein interaction with cytokine and cytokine receptor, hsa04151:PI3K-Akt signaling pathway, hsa04630:JAK-STAT signaling pathway, hsa05144:Malaria, hsa04510:Focal adhesion
III		✓	✓	hsa05322:Systemic lupus erythematosus, hsa04613:Neutrophil extracellular trap formation, hsa05034:Alcoholism, hsa05203:Viral carcinogenesis, hsa04217:Necroptosis
IV			✓	hsa04964:Proximal tubule bicarbonate reclamation, hsa05200:Pathways in cancer, hsa05219:Bladder cancer, hsa05224:Breast cancer, hsa04911:Insulin secretion, hsa04015:Rap1 signaling pathway, hsa04961:Endocrine and other factor-regulated calcium reabsorption, hsa00410:beta-Alanine metabolism, hsa04972:Pancreatic secretion, hsa04974:Protein digestion and absorption, hsa00910:Nitrogen metabolism, hsa04014:Ras signaling pathway
V	✓			hsa04940:Type I diabetes mellitus, hsa04917:Prolactin signaling pathway, hsa04668:TNF signaling pathway, hsa05323:Rheumatoid arthritis, hsa04913:Ovarian steroidogenesis, hsa05150:Staphylococcus aureus infection, hsa00650:Butanoate metabolism, hsa04080:Neuroactive ligand-receptor interaction, hsa04610:Complement and coagulation cascades, hsa00830:Retinol metabolism
VI		✓		hsa04929:GnRH secretion, hsa04024:cAMP signaling pathway, hsa04064:NF-kappa B signaling pathway, hsa05217:Basal cell carcinoma

Class I: the pathways enriched commonly in the three exposure groups.

Class II: the pathways enriched in the capsaicin and combination exposure groups.

Class III: the pathways enriched in mitoxantrone and combination exposure groups.

Class IV: the pathways enriched only in the combination exposure group.

Class V: the pathways enriched only in the capsaicin exposure group.

Class VI: the pathways enriched only in the mitoxantrone exposure group.

We examined the gene expression changes in the Rap1 signaling pathway and compared them with the exposure groups. The fold changes of *RASGRP3, PDGFB*, and *THBS1* expression in the combined exposure group are more than twice higher compared with the total fold changes of capsaicin and mitoxantrone exposure groups (Fig. 4d, Fig. S4 and Fig. S5). These results suggest that the combination synergistically accelerated the expression of these genes.

Finally, we examined transcription factors (TFs) enriched in the three genes (e.g., *RASGRP3, PDGFB*, and *THBS1*). TFs are identified by Fisher’s exact test using the Enricher analysis tool^56^. We selected the ChIP-x Enrichment Analysis (ChEA) database as a gene set library. The ChEA database contains putative targets for TFs extracted from publications on experimental profiling of TFs binding to DNA in mammalian cells^47^. The stem cell leukemia gene (SCL), also known as Tal-1^57^, is the most statistically enriched TF (Fig. S4). This suggests that capsaicin and mitoxantrone induce *RASGRP3, PDGFB*, and *THBS1* expression through SCL to inhibit cell survival (Fig. 4e).

## Discussion

We proposed a network-based trans-omics approach, SyndrumNET, to predict synergistic drug combinations for various human diseases. The originality of the method lies in its ability to identify drug combinations considering various biological processes, which was achieved by integrating multi-omics data representing different biological processes, such as static information on molecular interaction networks and dynamic information on drug transcriptomic responses. We emphasized transcriptional correlations between disease modules and drug modules using network propagation, to improve prediction accuracy. The method can be expanded to encompass additional diseases and tailored to specific gene signatures. By constructing gene expression profiles for novel drugs, our proposed method can be effectively employed for analyzing drug-specific response profiles. This method is also adaptable to any compounds with available response profiles. We predicted new drug combinations for CML using this method and validated the anticancer activity of predicted drug combination in vitro. We identified the underlying mode-of-action the drug synergy by microarray analysis at the pathway level. The proposed method will be useful for predicting synergistic drug combinations for various diseases.

We demonstrated that drug modules constructed by drug response genes tend to be in the neighborhood of associated diseases. Network-based methods have traditionally used drug target molecules to characterize drugs and construct drug modules^13,15,58,59^; however, few drugs have known target molecules. In addition, only a limited number of drugs have a sufficient number of target molecules to construct a drug module^16^. A recent study using a network-based method demonstrated that genes which respond to drugs and compounds are useful for predicting the efficacy of the drugs and compounds^60^. Therefore, it is reasonable to use drug response genes for constructing drug modules as an alternative to target molecules.

We used transcriptional correlations between disease modules and drug modules. This transcriptome-based method facilitates the systematic comparison of gene expression profiles that characterize the response to drugs and biological states of interest. The disadvantage of a transcriptome-based method is that correlation scores for disease–drug pairs tend to be low^11^. It is reported that most disease–drug pairs exhibit a weak transcriptional correlation when using gene expression of all protein-coding genes^14^. We found that modules were expanded by network propagation and the overlapping genes between expanded modules emphasized the transcriptomic correlation between the disease and drug module. This suggests that the expansion of modular genes using network propagation is effective for deterring transcriptional correlations between diseases and drugs. The network propagation method will contribute to the enhancement of prediction accuracy of the transcriptome-based method.

In this study, we focused on CML. Three discrete clinical stages are defined for CML: the chronic phase, the accelerated phase, and the blast crisis. Drugs known as tyrosine kinase inhibitors (TKIs) that target BCR-ABL are the standard treatment for CML^61^. Patients in the chronic phase achieve a 10-year survival rate of more than 90% with the TKIs. On the other hand, the prognosis of patients in the blast phase is poor, and treatments are limited. We used the K562 cell line derived from a patient in the blast phase for experimental validation to propose effective drug combinations for the blast phase of CML. We found that the combination of capsaicin and mitoxantrone exhibited synergistic effects on the CML cells. Neither capsaicin nor mitoxantrone targets the BCR-ABL fusion gene. In addition, neither drug module included BCR or ABL gene. The synergistic effects of the combination of capsaicin and mitoxantrone seems not be occurred through BCR-ABL inhibition and could be combined with standard therapy using TKIs. The synergistic effects of capsaicin and mitoxantrone seems to be occurred through the inhibition of DNA repair by mitoxantrone and the anticancer effect of capsaicin. Anticancer mechanisms are primarily related to induction of apoptosis and autophagy, reduced proliferation, as well as the inhibition of angiogenesis and metastasis^62^. Consistent with previous results, DEGs associated with capsaicin exposure were enriched in cancer pathways in our microarray analysis (Fig. 4). Mitoxantrone is known to be an inhibitor of topoisomerase II^63^. Thus, mitoxantrone may alter the expression of several genes, such as TFs and receptors, for capsaicin. The genes stimulated by capsaicin may alter the expression of downstream genes. Indeed, the expression of 1,313 genes were affected only by combination treatment (Fig. 4).

We detected the RAP1 signaling pathway as an important functional pathway exerting a synergistic effect in response to capsaicin and mitoxantrone. Our hypothesis is based on observations from our microarray analysis and previous observations about the function of genes in the RAP1 signaling pathway on cancer. RAP1, a member of the shelterin complex, has been implicated in cancer development^64^. For example, RAP1 activated by RasGRP3 increased cell migration and invasion in glioma cells^65^. From our microarray analysis, the expression of *RAP1* was not altered by combination exposure. On the other hand, *RASGRP3* was overexpressed by combination exposure (Fig. 4e and Fig. S8). Thus, it is possible that overexpression of *RASGRP3* by combined drug exposure activates *RAP1*.

THBS1, as a tumor suppressor gene, influences the growth of tumors by inhibiting angiogenesis and activating the transforming growth factor. THBS1 is weakly expressed in AML patients^66^, which is associated with a shorter survival time^66^. From our microarray analysis, *THBS1* expression was notably increased by combination exposure (Fig. 4e and Fig. S4). The results suggest that enhanced *THBS1* expression by combination exposure suppresses the proliferation of CML cells. Future experimental validation is needed to confirm the mode-of-action of the drug combination through the RAP1 signaling pathway.

We examined the mode-of-action of the synergistic drug combination in the context of transcriptome factors. We found that SCL was enriched in the promoter region of overexpressed genes by combination treatment, such as *RASGRP3, PDGFB*, and *THBS1*. Increased expression of SCL is associated with leukemia and poor prognosis of T-cell acute lymphoblastic leukemia^67^. Interestingly, the expression of SCL was slightly decreased in the drug combination group (Fig. S6). This suggests that the suppressed expression of SCL may contribute to the synergistic effects of the combination.

We opted for microarray technology to understand the mode of action of the synergistic effect of capsaicin and mitoxantrone over RNA-seq analysis due to our emphasis on assessing the expression levels of coding regions in genes with known functions. Microarray technology offers advantages in terms of speed, simplicity, and affordability and requires minimal RNA input for the detection of the expression levels of known genes. While acknowledging the inherent differences between RNA-Seq and microarray technologies, it is worth noting that previous studies^68,69^ have reported a notable overlap (approximately 70%–80%) in differentially expressed genes identified by both methods, with a Spearman’s correlation ranging from 0.7 to 0.8. These findings imply that transitioning from microarray to RNA-Seq may not substantially alter the results. Furthermore, the primary distinction between microarray and RNA-Seq technologies lies in their gene detection capabilities: microarrays quantify a predetermined set of known genes (e.g., mRNA), whereas RNA-Seq can sequence all RNAs present, including those with unknown functions like miRNA and non-coding RNA. Our analysis of gene expression profiles aimed to uncover the mechanism behind the observed synergistic effect. KEGG pathway analysis excludes genes with unknown functions due to current limitations in pathway databases and analytical frameworks. Given these limitations, we argue that microarray data suffice for this study’s objectives because our focus is on elucidating mechanisms through known genes and their functional relationships in established pathways. However, we acknowledge the potential advantages of RNA-Seq, such as its broader dynamic range and ability to identify expressions of genes with unknown functions, including miRNA and non-coding RNA. The use of RNA-Seq offers significant analytical benefits, especially when the aim is to elucidate novel pathways or characterize genes with previously unknown functions.

In this study, we employed a human molecular interaction network created from various public databases. This network includes 13,377 proteins and 235,123 interactions, covering 65% of the genes responsible for human protein coding (Supplementary Data S2). It is worth noting that protein interaction databases undergo continuous updates. Since our methodology can be adjusted to accommodate new datasets, utilizing updated protein interaction network data holds promise for enhancing the accuracy and comprehensiveness of drug combination discovery. Developing predictive models based on the most recent protein interaction network data stands as a vital avenue for future research.

One limitation of the proposed method is that it does not consider disease subtypes. For example, the World Health Organization (WHO) classified AML into 25 subtypes, including two provisional entities, which differ in prognosis and treatment^70^; however, the genetic features of some subtypes remain unclear^71^. In addition, there may be few differences in genetic characteristics between the subtypes. According to the WHO classification, AML with *RUNX1* mutation, AML with *NPM1* mutation, and AML with biallelic *CEBPA* mutations are considered distinct categories^70^. In our proposed method, genetic features are used to define disease modules. A lack of variation in genetic features among subtypes results in reduced diversity in the predicted results and prediction accuracy. To predict effective drug combinations for each subtype, genetic data for the individual subtypes is needed. A recent study succeeded in predicting drug combinations for melanoma subtypes by considering transcriptome correlations and network centralities of genes between disease subtypes and drugs^72^. The incorporation of variation in genetic features could establish a prediction method for individual disease subtypes.

### Supplementary information

Supplementary Information

Description of Additional Supplementary Files

Supplementary Data 1

Supplementary Data 2

Supplementary Data 3

Supplementary Data 4

Supplementary Data 5

Supplementary Data 6

Supplementary Data 7

Supplementary Data 8

## Data Availability

We have only use existing publicly available datasets for analysis. The source databases for constructing human molecular interactions are summarized in from Supplementary Data S1. Human molecular interactions constructed in this study are available in Supplementary Data S2. A set of disease susceptibility genes and drug related genes are described in Supplementary Data S3 and S4. Gene expression signatures for 79 diseases with 14,804 genes are in the CREEDS database^24^. Gene expression signatures for hypertension can be retrieved from GEO (GSE24752, and GSE75360). Drug-induced gene expression profiles can be obtained from the LINCS Program L1000 mRNA profiling assay^49^. Drug combinations with synergistic effects are listed in Supplementary Data S6. The prediction results by SyndrumNET can be seen in Supplementary Data S7. Our microarray data can be found at GSE254052. The source data for Fig. 2a–c are in Supplementary Data Set 1^73^, Supplementary Data Set 2^74^, and Supplementary Data Set 3^75^, respectively. The source data for Fig. 3a–c are in Supplementary Data Set 4^76^, Supplementary Data Set 5^77^, and Supplementary Data S2, S3, and S4, respectively. The source data of Figs. 4a, b, d are in GSE254052 and Supplementary Data S8, and for 4c is in Supplementary Data Set 6^78^. All other data are available from the corresponding author on reasonable request.

## Code availability

The functions used for our analyses are available at igraph (1.2.6), biomaRt (4.0.3), lsa (0.73.2), ROCR (v.1.0-11), and clusterProfiler (v4.4.4). The code for network propagation is available at: https://github.com/kztakemoto/network_propagation. The scripts for calculating prediction score and AUC are in Supplementary Data Set 7^35^.

## References

[1] Mokhtari, R. B. et al. Combination therapy in combating cancer. *Oncotarget* **8**, 38022–38043 (2017). 28410237 10.18632/oncotarget.16723 PMC5514969

[2] Doroshow, J. H. & Simon, R. M. On the design of combination cancer therapy. *Cell* **171**, 1476–1478 (2017). 29245008 10.1016/j.cell.2017.11.035 PMC7422640

[3] Gradman, A. H., Basile, J. N., Carter, B. L. & Bakris, G. L. Combination therapy in hypertension. *J. Clin. Hypertens.* **13**, 146–154 (2011). 10.1111/j.1751-7176.2010.00397.x PMC8673364 21366845

[4] Das, P., Delost, M. D., Qureshi, M. H., Smith, D. T. & Njardarson, J. T. A survey of the structures of US FDA approved combination drugs. *J. Med. Chem.* **62**, 4265–4311 (2019). 30444362 10.1021/acs.jmedchem.8b01610

[5] FDA. Fact Sheet: FDA at a Glance. *U.S. FOOD & DRUG ADMINISTRATION From the OFFICE OF THE COMMISSIONER* November (2021). Available at: https://www.fda.gov/about-fda/fda-basics/fact-sheet-fda-glance. (Accessed: 31st October 2022).

[6] Kong, W. et al. Systematic review of computational methods for drug combination prediction. *Comput. Struct. Biotechnol. J.* **20**, 2807–2814 (2022). 35685365 10.1016/j.csbj.2022.05.055 PMC9168078

[7] Zhao, X. M. et al. Prediction of drug combinations by integrating molecular and pharmacological data. *PLOS Comput. Biol.* **7**, e1002323 (2011). 22219721 10.1371/journal.pcbi.1002323 PMC3248384

[8] Iwata, H., Sawada, R., Mizutani, S., Kotera, M. & Yamanishi, Y. Large-scale prediction of beneficial drug combinations using drug efficacy and target profiles. *J. Chem. Inf. Model.* **55**, 2705–2716 (2015). 26624799 10.1021/acs.jcim.5b00444

[9] Celebi, R. O., Bear Don’t Walk, R., Movva, S., Alpsoy, S. & Dumontier, M. In-silico prediction of synergistic anti-cancer drug combinations using multi-omics data. *Sci. Rep.* **9**, 8949 (2019). 31222109 10.1038/s41598-019-45236-6 PMC6586895

[10] Jin, W. et al. Deep learning identifies synergistic drug combinations for treating COVID-19. *Proc. Natl Acad. Sci. USA* **118**, e2105070118 (2021). 34526388 10.1073/pnas.2105070118 PMC8488647

[11] Stathias, V. et al. Drug and disease signature integration identifies synergistic combinations in glioblastoma. *Nat. Commun.* **9**, 5315 (2018). 30552330 10.1038/s41467-018-07659-z PMC6294341

[12] Li, X., Qin, G., Yang, Q., Chen, L. & Xie, L. Biomolecular Network-Based Synergistic Drug Combination Discovery. *Biomed. Res. Int.* **2016**, 1–11 (2016). 10.1155/2016/8518945 PMC5116515 27891522

[13] Cheng, F., Kovács, A. & Barabási, A. Network-based prediction of drug combinations. *Nat. Commun.* **10**, 1197 (2019). 30867426 10.1038/s41467-019-09186-x PMC6416394

[14] Iwata, M. et al. Regulome-based characterization of drug activity across the human diseasome. *npj Syst. Biol. Appl.* **8**, 44 (2022). 36344521 10.1038/s41540-022-00255-4 PMC9640590

[15] Guney, E., Menche, J., Vidal, M. & Barábasi, A.-L. Network-based in silico drug efficacy screening. *Nat. Commun.* **7**, 10331 (2016). 26831545 10.1038/ncomms10331 PMC4740350

[16] Yıldırım, M. A., Goh, K.-I., Cusick, M. E., Barabási, A.-L. & Vidal, M. Drug—target network. *Nat. Biotechnol.* **25**, 1119–1126 (2007). 17921997 10.1038/nbt1338

[17] Luck, K. et al. A reference map of the human binary protein interactome. *Nature* **580**, 402–408 (2020). 32296183 10.1038/s41586-020-2188-x PMC7169983

[18] Giurgiu, M. et al. CORUM: the comprehensive resource of mammalian protein complexes—2019. *Nucleic Acids Res.* **47**, D559–D563 (2019). 30357367 10.1093/nar/gky973 PMC6323970

[19] Hornbeck, P. V. et al. PhosphoSitePlus: a comprehensive resource for investigating the structure and function of experimentally determined post-translational modifications in man and mouse. *Nucleic Acids Res.* **40**, D261–70 (2012). 22135298 10.1093/nar/gkr1122 PMC3245126

[20] Shimizu, Y., Hattori, M., Goto, S. & Kanehisa, M. Generalized reaction patterns for prediction of unknown enzymatic reactions. *Genome Inf.* **20**, 149–58 (2008). 19425130

[21] Fazekas, D. et al. SignaLink 2 – a signaling pathway resource with multi-layered regulatory networks. *BMC Syst. Biol.* **7**, 7 (2013). 23331499 10.1186/1752-0509-7-7 PMC3599410

[22] Breuer, K. et al. InnateDB: Systems biology of innate immunity and beyond - Recent updates and continuing curation. *Nucleic Acids Res.* **41**, D1228–D1233 (2013). 23180781 10.1093/nar/gks1147 PMC3531080

[23] Meyer, M. J., Das, J., Wang, X. & Yu, H. INstruct: A database of high-quality 3D structurally resolved protein interactome networks. *Bioinformatics* **29**, 1577–1579 (2013). 23599502 10.1093/bioinformatics/btt181 PMC3673217

[24] Wang, Z. et al. Extraction and analysis of signatures from the Gene Expression Omnibus by the crowd. *Nat. Commun.* **7**, 12846 (2016). 27667448 10.1038/ncomms12846 PMC5052684

[25] Edgar, R. Gene Expression Omnibus: NCBI gene expression and hybridization array data repository. *Nucleic Acids Res.* **30**, 207–210 (2002). 11752295 10.1093/nar/30.1.207 PMC99122

[26] Amberger, J. S., Bocchini, C. A., Schiettecatte, F., Scott, A. F. & Hamosh, A. OMIM.org: Online Mendelian Inheritance in Man (OMIM®), an Online catalog of human genes and genetic disorders. *Nucleic Acids Res.* **43**, D789–D798 (2015). 25428349 10.1093/nar/gku1205 PMC4383985

[27] Landrum, M. J. et al. ClinVar: Public archive of relationships among sequence variation and human phenotype. *Nucleic Acids Res.* **42**, D980–5 (2014). 24234437 10.1093/nar/gkt1113 PMC3965032

[28] Welter, D. et al. The NHGRI GWAS Catalog, a curated resource of SNP-trait associations. *Nucleic Acids Res.* **42**, D1001–D1006 (2014). 24316577 10.1093/nar/gkt1229 PMC3965119

[29] Denny, J. C. et al. Systematic comparison of phenome-wide association study of electronic medical record data and genome-wide association study data. *Nat. Biotechnol.* **31**, 1102–1111 (2013). 24270849 10.1038/nbt.2749 PMC3969265

[30] Li, M. J. et al. GWASdb: A database for human genetic variants identified by genome-wide association studies. *Nucleic Acids Res.* **40**, D1047–D1054 (2012). 22139925 10.1093/nar/gkr1182 PMC3245026

[31] Pinero, J. et al. DisGeNET: A discovery platform for the dynamical exploration of human diseases and their genes. *Database* **2015**, bav028 (2015). 25877637 10.1093/database/bav028 PMC4397996

[32] Durinck, S. et al. BioMart and Bioconductor: A powerful link between biological databases and microarray data analysis. *Bioinformatics* **21**, 3439–3440 (2005). 16082012 10.1093/bioinformatics/bti525

[33] Subramanian, A. et al. A Next Generation Connectivity Map: L1000 Platform and the First 1,000,000 Profiles. *Cell* **171**, 1437–1452.e17 (2017). 29195078 10.1016/j.cell.2017.10.049 PMC5990023

[34] Berenger, F., Coti, C. & Zhang, K. Y. J. PAR: A PARallel And Distributed Job Crusher. *Bioinformatics*. 10.1093/bioinformatics/btq542 (2010) 10.1093/bioinformatics/btq542 20870644

[35] Iida, M. Code for A network-based trans-omics approach for predicting synergistic drug combinations. 10.6084/m9.figshare.25735206. (2024) 10.1038/s43856-024-00571-2 PMC11286857 39075184

[36] Iida, M., Iwata, M. & Yamanishi, Y. Network-based characterization of disease-disease relationships in terms of drugs and therapeutic targets. *Bioinformatics* **36**, i516–i524 (2020). 32657408 10.1093/bioinformatics/btaa439 PMC7355285

[37] Kanehisa, M., Goto, S., Furumichi, M., Tanabe, M. & Hirakawa, M. KEGG for representation and analysis of molecular networks involving diseases and drugs. *Nucleic Acids Res.* **38**, D355–D360 (2010). 19880382 10.1093/nar/gkp896 PMC2808910

[38] Kotera, M. et al. KCF-S: KEGG Chemical Function and Substructure for improved interpretability and prediction in chemical bioinformatics. *BMC Syst. Biol.* **7**, S2 (2013). 24564846 10.1186/1752-0509-7-S6-S2 PMC4029371

[39] Aoto, Y. et al. Time-series analysis of tumorigenesis in a murine skin carcinogenesis model. *Sci. Rep.* **8**, 12994 (2018). 30158594 10.1038/s41598-018-31349-x PMC6115443

[40] Vanunu, O., Magger, O., Ruppin, E., Shlomi, T. & Sharan, R. Associating genes and protein complexes with disease via network propagation. *PLoS Comput. Biol.* **6**, e1000641 (2010). 20090828 10.1371/journal.pcbi.1000641 PMC2797085

[41] Csardi, G. & Nepusz, T. The igraph software package for complex network research. *InterJournal* Complex Sy, 1695 (2006).

[42] Liu, H. et al. DrugCombDB: a comprehensive database of drug combinations toward the discovery of combinatorial therapy. *Nucleic Acids Res.* **48**, 871 (2020). 10.1093/nar/gkz1007 PMC7145671 31665429

[43] Bliss, C. I. The toxicity of poisons applied jointly 1. *Ann. Appl. Biol.* **26**, 585–615 (1939). 10.1111/j.1744-7348.1939.tb06990.x

[44] Loewe, S. & Muischnek, H. Effect of combinations: Mathematical basis of the problem. *Arch. Exp. Pathol. Pharmakol.* **114**, 313–326 (1926). 10.1007/BF01952257

[45] Sherman, B. T. et al. DAVID: a web server for functional enrichment analysis and functional annotation of gene lists (2021 update). *Nucleic Acids Res.* **50**, W216–W221 (2022). 35325185 10.1093/nar/gkac194 PMC9252805

[46] Wu, T. et al. clusterProfiler 4.0: A universal enrichment tool for interpreting omics data. *Innov* **2**, 100141 (2021). 10.1016/j.xinn.2021.100141 PMC8454663 34557778

[47] Lachmann, A. et al. ChEA: Transcription factor regulation inferred from integrating genome-wide ChIP-X experiments. *Bioinformatics* **26**, 2438–2444 (2010). 20709693 10.1093/bioinformatics/btq466 PMC2944209

[48] Menche, J. et al. Disease networks. Uncovering disease-disease relationships through the incomplete interactome. *Science* **347**, 1257601 (2015). 25700523 10.1126/science.1257601 PMC4435741

[49] Koleti, A. et al. Data Portal for the Library of Integrated Network-based Cellular Signatures (LINCS) program: Integrated access to diverse large-scale cellular perturbation response data. *Nucleic Acids Res.* **46**, D558–D566 (2018). 29140462 10.1093/nar/gkx1063 PMC5753343

[50] Choi, S. W. & Ho, C. K. Antioxidant properties of drugs used in Type 2 diabetes management: could they contribute to, confound or conceal effects of antioxidant therapy? *Redox Rep.* **23**, 1–24 (2018). 28514939 10.1080/13510002.2017.1324381 PMC6748682

[51] Park, S. J. et al. Topotecan-based combination chemotherapy in patients with transformed chronic myelogenous leukemia and advanced myelodysplastic syndrome. *Korean J. Intern. Med.* **15**, 122–126 (2000). 10992724 10.3904/kjim.2000.15.2.122 PMC4531761

[52] He, L. et al. Methods for high-throughput drug combination screening and synergy scoring. *Methods Mol. Biol.* **1711**, 351–398 (2018). 29344898 10.1007/978-1-4939-7493-1_17 PMC6383747

[53] Looi, C. K., Hii, L. W., Ngai, S. C., Leong, C. O. & Mai, C. W. The role of Ras-associated Protein 1 (Rap1) in cancer: bad actor or good player? *Biomedicines* **8**, 334 (2020). 32906721 10.3390/biomedicines8090334 PMC7555474

[54] Wittchen, E. S. et al. Rap1 GTPase inhibits leukocyte transmigration by promoting endothelial barrier function. *J. Biol. Chem.* **280**, 11675–11682 (2005). 15661741 10.1074/jbc.M412595200

[55] Katagiri, K. et al. Rap1 is a potent activation signal for leukocyte function-associated antigen 1 distinct from protein kinase C and phosphatidylinositol-3-OH kinase. *Mol. Cell. Biol.* **20**, 1956–1969 (2000). 10688643 10.1128/MCB.20.6.1956-1969.2000 PMC110813

[56] Chen, E. Y. et al. Enrichr: Interactive and collaborative HTML5 gene list enrichment analysis tool. *BMC Bioinf.* **14**, 128 (2013). 10.1186/1471-2105-14-128 PMC3637064 23586463

[57] Barton, L. M. et al. Regulation of the stem cell leukemia (SCL) gene: A tale of two fishes. *Proc. Natl Acad. Sci. USA* **98**, 6747–6752 (2001). 11381108 10.1073/pnas.101532998 PMC34424

[58] Barabási, A. L., Gulbahce, N. & Loscalzo, J. Network medicine: A network-based approach to human disease. *Nat. Rev. Genet.* **12**, 56–68 (2011). 21164525 10.1038/nrg2918 PMC3140052

[59] Cheng, F. et al. Network-based approach to prediction and population-based validation of in silico drug repurposing. *Nat. Commun.* **9**, 2691 (2018). 30002366 10.1038/s41467-018-05116-5 PMC6043492

[60] do Valle, I. F. et al. Network medicine framework shows that proximity of polyphenol targets and disease proteins predicts therapeutic effects of polyphenols. *Nat. Food* **2**, 143–155 (2021). 37117448 10.1038/s43016-021-00243-7

[61] American Cancer Society. Targeted Therapies for Chronic Myeloid Leukemia. Available at: https://www.cancer.org/cancer/chronic-myeloid-leukemia/treating/targeted-therapies.html (2023). (Accessed: 1st March 2023).

[62] Zhang, S., Wang, D., Huang, J., Hu, Y. & Xu, Y. Application of capsaicin as a potential new therapeutic drug in human cancers. *J. Clin. Pharm. Ther.* **45**, 16–28 (2020). 31545523 10.1111/jcpt.13039

[63] Osheroff, N., Corbett, A. H. & Robinson, M. J. Mechanism of action of topoisomerase II-targeted antineoplastic drugs. *Adv. Pharmacol.* **29**, 105–126 (1994). 10.1016/S1054-3589(08)61134-5 8996604

[64] Deregowska, A. & Wnuk, M. RAP1/TERF2IP-a multifunctional player in cancer development. *Cancers (Basel)* **13**, 5970 (2021). 34885080 10.3390/cancers13235970 PMC8657031

[65] Lee, H. K. et al. RasGRP3 regulates the migration of glioma cells via interaction with Arp3. *Oncotarget* **6**, 1850–1864 (2015). 25682201 10.18632/oncotarget.2575 PMC4359336

[66] Zhu, L. et al. THBS1 is a novel serum prognostic factors of acute myeloid leukemia. *Front. Oncol.* **9**, 1567 (2020). 32117788 10.3389/fonc.2019.01567 PMC7020255

[67] Porcher, C., Chagraoui, H. & Kristiansen, M. S. SCL/TAL1: A multifaceted regulator from blood development to disease. *Blood* **129**, 2051–2060 (2017). 28179281 10.1182/blood-2016-12-754051

[68] Xu, X. et al. Parallel comparison of Illumina RNA-Seq and Affymetrix microarray platforms on transcriptomic profiles generated from 5-aza-deoxy-cytidine treated HT-29 colon cancer cells and simulated datasets. *BMC Bioinforma.* **14**, S1 (2013). 10.1186/1471-2105-14-S9-S1 PMC3697991 23902433

[69] Rao, M. S. et al. Comparison of RNA-Seq and microarray gene expression platforms for the toxicogenomic evaluation of liver from short-term rat toxicity studies. *Front. Genet.* **9**, 636 (2019). 30723492 10.3389/fgene.2018.00636 PMC6349826

[70] Narayanan, D. & Weinberg, O. K. How I investigate acute myeloid leukemia. *Int. J. Lab. Hematol.* **42**, 3–15 (2020). 31820579 10.1111/ijlh.13135

[71] Rose, D. et al. Subtype-specific patterns of molecular mutations in acute myeloid leukemia. *Leukemia* **31**, 11–17 (2017). 27285584 10.1038/leu.2016.163

[72] Regan-Fendt, K. E. et al. Synergy from gene expression and network mining (SynGeNet) method predicts synergistic drug combinations for diverse melanoma genomic subtypes. *npj Syst. Biol. Appl.* **5**, 6 (2019). 30820351 10.1038/s41540-019-0085-4 PMC6391384

[73] Iida, M. Figure 2a Distribution of average network-based proximity (PQAB) between a query disease module (Q) and drug modules (A and B). 10.6084/m9.figshare.25699275.v1.

[74] Iida, M. Figure 2b Distribution of the number of overlapped genes between a query disease module (Q) and individual drug modules (A or B). 10.6084/m9.figshare.25699356.v1.

[75] Iida, M. Figure 2c Distribution of average absolute transcriptional correlation coefficients between a query disease module (Q) and drug modules (A and B). 10.6084/m9.figshare.25699434.v1.

[76] Iida, M. Figure 3a Enriched functional pathways in the CML module, and the capsaicin and mitoxantrone modules in the method with network propagation (SyndrumNET). 10.6084/m9.figshare.25699446.v1.

[77] Iida, M. Figure 3b Distribution of the coverage of enriched pathways between the CML module and drug modules. 10.6084/m9.figshare.25705947.

[78] Iida, M. Figure 4c The significance of the pathways enriched only in the combination exposure group. 10.6084/m9.figshare.25705959.v1.

