# Supplementary material for: A network-based trans-omics approach for predicting synergistic drug combinations
Source: Commun Med (Lond). 2024 Jul 29;4:154. doi: 10.1038/s43856-024-00571-2 (PMC11286857; doi:10.1038/s43856-024-00571-2)
Supplement: Supplementary file 1 — Supplementary Information [file 43856_2024_571_MOESM1_ESM.pdf]

Supplementary Information for

A network-based trans-omics approach for predicting synergistic drug combinations

Midori Iida[1,2], Yurika Kuniki[3], Kenta Yagi[3,4], Mitsuhiro Goda[3,5], Satoko Namba[2,6], Jun-ichi Takeshita[7], Ryusuke Sawada[2,8], Michio Iwata[2], Yoshito Zamami[3,9], Keisuke Ishizawa[3,4,5], Yoshihiro Yamanishi[2,6]*

Affiliations

1) Department of Physics and Information Technology, Kyushu Institute of Technology, Iizuka, Fukuoka 820-8502, Japan

2) Department of Bioscience and Bioinformatics, Kyushu Institute of Technology, Iizuka, Fukuoka 820-8502, Japan

3) Department of Clinical Pharmacology and Therapeutics, Tokushima University Graduate School of Biomedical Sciences, Kuramoto-Cho, Tokushima 770-8503, Japan

4) Clinical Research Center for Developmental Therapeutics, Tokushima University Hospital, Kuramoto-cho, Tokushima 770-8503, Japan

5) Department of Pharmacy, Tokushima University Hospital, Kuramoto-Cho, Tokushima 770- 8503, Japan

6) Department of Complex Systems Science, Graduate School of Informatics, Nagoya University, Chikusa, Nagoya, Aichi 464-8601, Japan

7) Research Institute of Science for Safety and Sustainability, National Institute of Advanced Industrial Science and Technology (AIST), Tsukuba, Ibaraki 305-8569, Japan

8) Department of Pharmacology, Okayama University Graduate School of Medicine, Dentistry, and Pharmaceutical Sciences, Okayama, Okayama, 700-8558, Japan

9) Department of Pharmacy, Okayama University Hospital, Kita-ku, Okayama 700-8558, Japan

*****Corresponding author: Email: yamanishi@i.nagoya-u.ac.jp

This PDF file includes:
Supplementary Text, Figures S1 to S6.

Supplementary Note 1

Supplemental Methods

Construction of disease-specific gene expression profiles

The CREEDS database lacked disease-specific gene expression signature for hypertension. Therefore, we constructed disease-specific gene expression signatures for hypertension according to the method from CREEDS database [1]. First, gene expression datasets for hypertension were retrieved from GEO database: GSE24752, which included 54,675 features from 6 samples, and GSE75360, which included 47,231 features from 21 samples (accessed on November 9, 2022). GSE75360 consists of data from two distinct racial groups: African Americans and Caucasians. Using the *chdirAnalysis* function in the *GeoDE* library in R, we determined disease-specific gene expression levels and differential significance relative to a healthy cohort. Given the racial diversity in GSE75360, we performed the analyses separately for African Americans and Caucasians. This yielded three distinct characteristic direction coefficients datasets: GSE24752 with its 54,675 features, GSE75360 for African Americans bearing 47,231 features, and GSE75360 for Caucasians, also with 47,231 features. From each dataset, we selected the top 600 features by the magnitude of their characteristic direction coefficients and averaged the coefficients by features. Then, we converted the features ID into ENTREZ gene ID. We treated the averaged coefficients as gene expression signature for hypertension. As the result, we obtained hypertension-specific gene expression signature for 1,479 genes. To align the genes from the disease-specific gene expression signature in the CREEDS database with the hypertension-specific gene expression signature constructed from GEO, we directly used the

expression signature of 1,089 genes out of the 1,479 genes specific to hypertension and set the values for the remaining 13,715 genes to 0. Finally, we obtained 14,804 genes for 6 diseases based on the gene expression profiles.

Construction of drug response gene expression profiles

Drug-induced gene expression profiles were sourced from the LINCS Program L1000 mRNA profiling assay (http://www.lincsproject.org). This program cataloged the gene expression profiles for 978 landmark genes, termed L1000 genes, at various post-treatment intervals—3, 6, 24, 48, and 144 hours—and across a range of concentrations using diverse human cell lines. The LINCS provides five distinct data processing levels:

Level 1: Raw unprocessed flow cytometry data from Luminex (LXB)

Level 2: Gene expression values per 1000 genes after deconvolution (GEX)

Level 3: Quantile-normalized gene expression profiles of landmark genes and imputed transcripts (Q2NORM or INF)

Level 4: Gene signatures computed using z-scores relative to the plate population as control (ZSPCINF) or relative to the plate vehicle control (ZSVCINF)

Level 5: Differential gene expression signatures without experimental condition replicates.

In the level 5 dataset, gene expression profiles for 20,547 compounds are cataloged. Within this dataset, the category of perturbagen types using small-molecule compounds is labeled as “trt_cp”.

From the “trt_cp” category, we extracted gene expression data corresponding to 1,488 drugs. We then averaged the gene expression of each drug across experimental conditions, such as post-treatment intervals, the concentration of the drug, and cell lines. Details on these 1,488 drugs, including their names and efficacies, are provided in Data S 3.

Supplementary References

1. Wang, Z. *et al.* Extraction and analysis of signatures from the Gene Expression Omnibus by the crowd. *Nat. Commun.* **7**, 12846 (2016).

Supplementary Materials

Fig.S1. The network-based proximity between a query disease module (Q) and individual drug modules with and without known synergistic effects using the average shortest path length.

“Unknown” indicates the network-based proximity between a query disease module and drug modules with unknown effects (AML: 1,106,100 pairs, CML: 1,106,103 pairs, colorectal cancer: 1,105,598 pairs, asthma: 1,106,327 pairs, type 2 Diabetes: 1,106,324 pairs, hypertension: 1,106,318 pairs). “Known” indicates the network-based proximity between a query disease module and drug modules with known synergistic

effects (AML: 228 pairs, CML: 225 pairs, colorectal cancer: 730 pairs, asthma: 1 pair, type 2 Diabetes: 4 pairs, hypertension: 10 pairs). Center line, median; box limits, upper and lower quartiles; whiskers, 1.5x interquartile range; white points, mean; black points, outliers.

Fig.S2. The distribution of transcriptional correlation confidence and number of overlapping genes between a query disease module and the drug modules.

Upper and lower whiskers indicate standard deviations, and points indicate the mean. (a) The average transcriptional correlation confidence between a query disease module and drug modules before propagation. (b) The number of overlapping genes between a query disease module and the drug modules before propagation. (c) The average transcriptional correlation confidence between a query disease module and the drug modules after propagation. (d) The number of overlapping genes between a query disease module and the drug modules after propagation.

Fig.S3 Enriched functional pathways in the CML module, and the capsaicin and mitoxantrone modules in the method without network propagation (Syndrum) and the method with network propagation (SyndrumNET).

Purple and light purple colors indicate CML. Green and light green colors indicate capsaicin. Yellow and light-yellow colors indicate mitoxantrone. The p-value

calculated by Fisher exact test using “clusterProfiler”. The sample number is 8,772 genes. The size of points reflects the p-value of the enrichment analysis.

Fig.S4 Log_2 fold-change of genes in the Ras1 pathway.

Green, yellow, and cyan represent capsaicin, mitoxantrone and combination exposure, respectively. *ADCY6*;adenylate cyclase 6 (112), *ADCY9*;adenylate cyclase 9 (115), *AKT3*;AKT serine/threonine kinase 3 (10000), *ARAP3*;ArfGAP with RhoGAP domain, ankyrin repeat and PH domain 3 (64411), *DRD2*;dopamine receptor D2 (1813), *EGF*;epidermal growth factor (1950), *FARP2*;FERM, ARH/RhoGEF and pleckstrin domain protein 2 (9855), *FGFR3*;fibroblast growth factor receptor 3 (2261), *HRAS*;HRas proto-oncogene, GTPase (3265), *IGF1*;insulin like growth factor 1 (3479), *INSR*;insulin receptor (3643), *ITGAM*;integrin subunit alpha M (3684), *ITGB3*;integrin subunit beta 3 (3690), *LPAR1*;lysophosphatidic acid receptor 1 (1902), *LPAR5*;lysophosphatidic acid receptor 5 (57121), *MAGI1*;membrane associated guanylate kinase, WW and PDZ domain containing 1 (9223), *MET*;MET proto-oncogene, receptor tyrosine kinase (4233), *NGFR*;nerve growth factor receptor (4804), *PARD6A*;par-6 family cell polarity regulator alpha (50855), *PDGFB*;platelet derived growth factor subunit B (5155), *PFN4*;profilin family member 4 (375189), *RASGRP2*;RAS guanyl releasing protein 2 (10235), *RASGRP3*;RAS guanyl releasing protein 3 (25780), *RRAS*;RAS related (6237),

THBS1;thrombospondin 1 (7057), *VAV1*;vav guanine nucleotide exchange factor 1 (7409), and *VEGFD*;vascular endothelial growth factor D (2277).

Fig.S5 The synergistically regulated genes mapped to the RAP1 signaling pathway.

Green color shows proteins in RAP1 signaling pathway. PDGFB, RASGPR3 and THBS1 are highlighted in red color letter and yellow background. The official names of these genes are described in fig.4 legend.

Fig.S6 The expression levels of SCL in each exposure condition.

Cyan represents the combination exposure group. Green represents the capsaicin exposure group. Yellow represents the mitoxantrone exposure group.

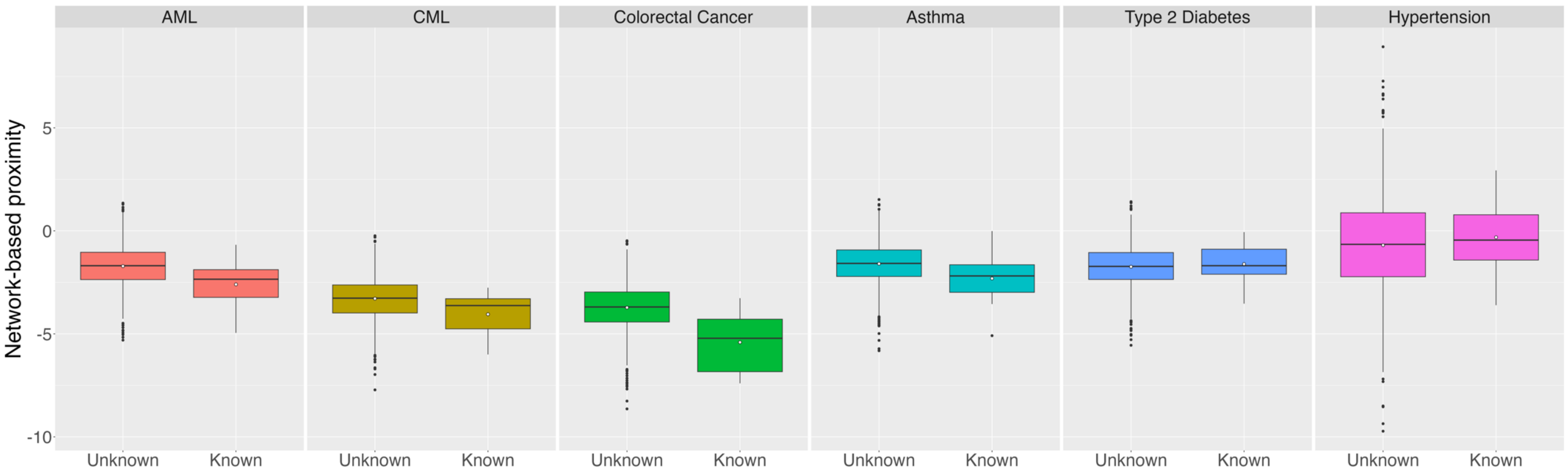

Fig.S1. The network-based proximity between a query disease module and individual drug modules with and without known synergistic effects using the average shortest path length.

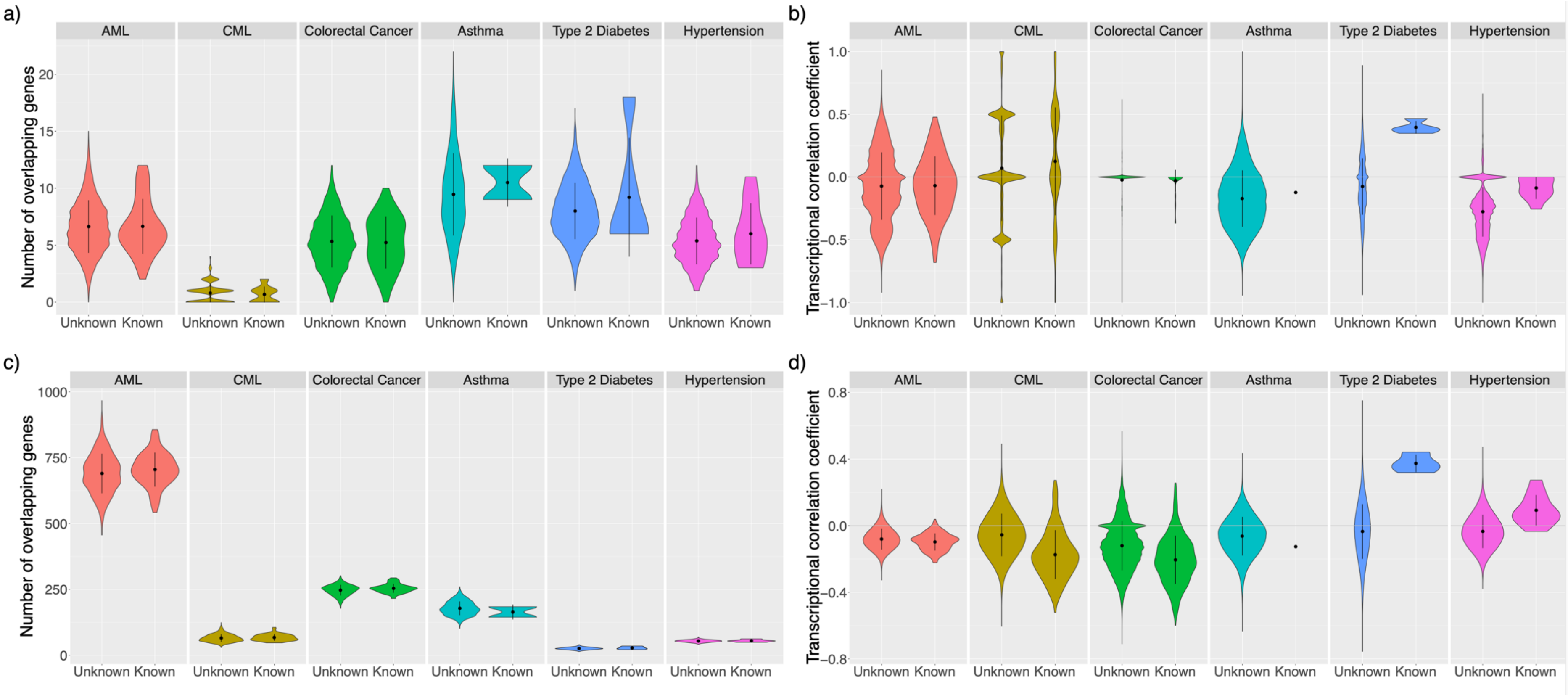

Fig.S2. The distribution of transcriptional correlation confidence and number of overlapping genes between a query disease module and the drug modules.

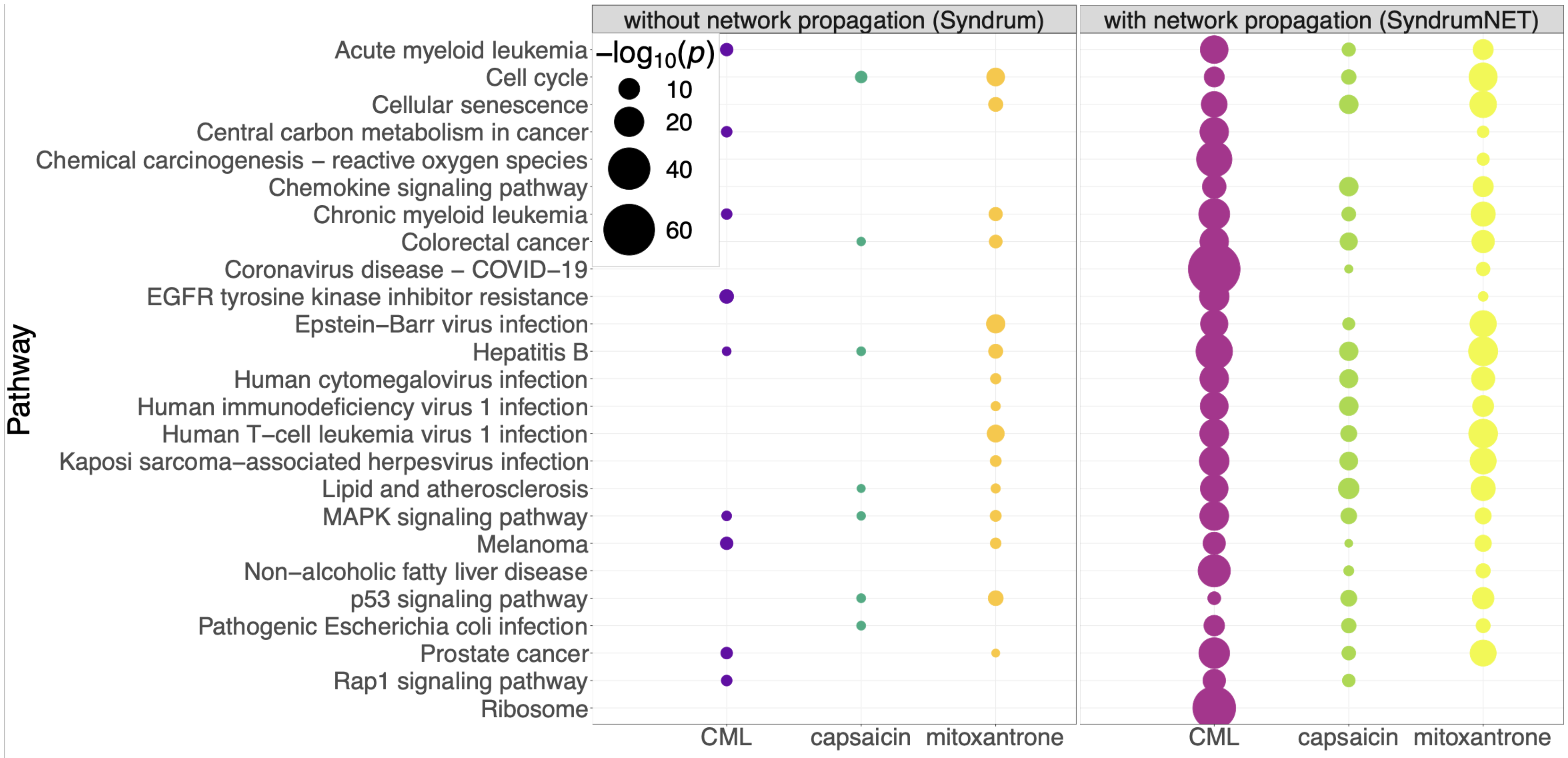

Fig.S3 Enriched functional pathways in the CML module, and the capsaicin and mitoxantrone modules in the method without network propagation (Syndrum) and the method with network propagation (SyndrumNET).

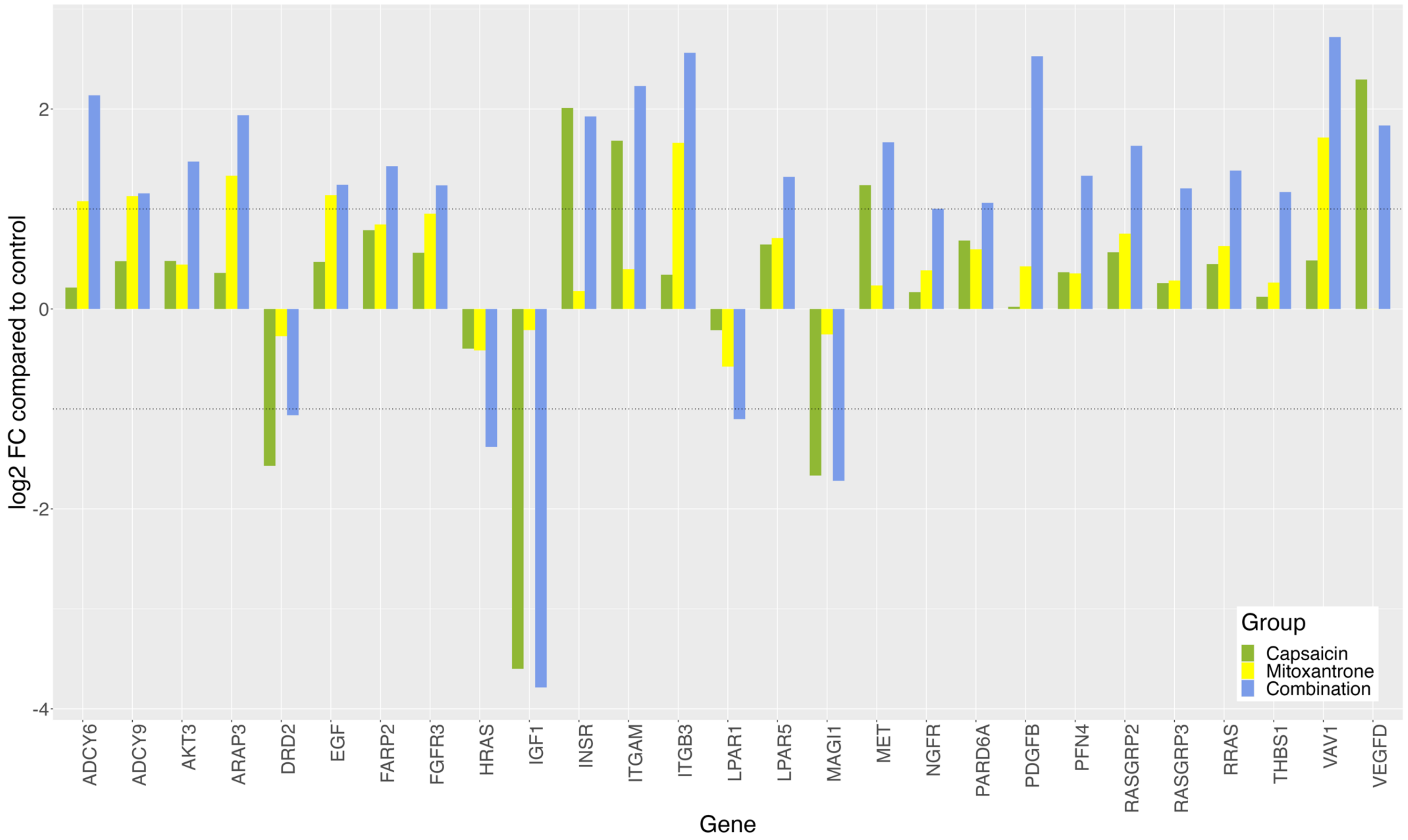

165

166 **Fig.S4.** Log$_2$ fold-change of genes in the Ras1 pathway. Green, yellow, and cyan represent capsaicin, mitoxantrone and combination
167 exposure, respectively.

168

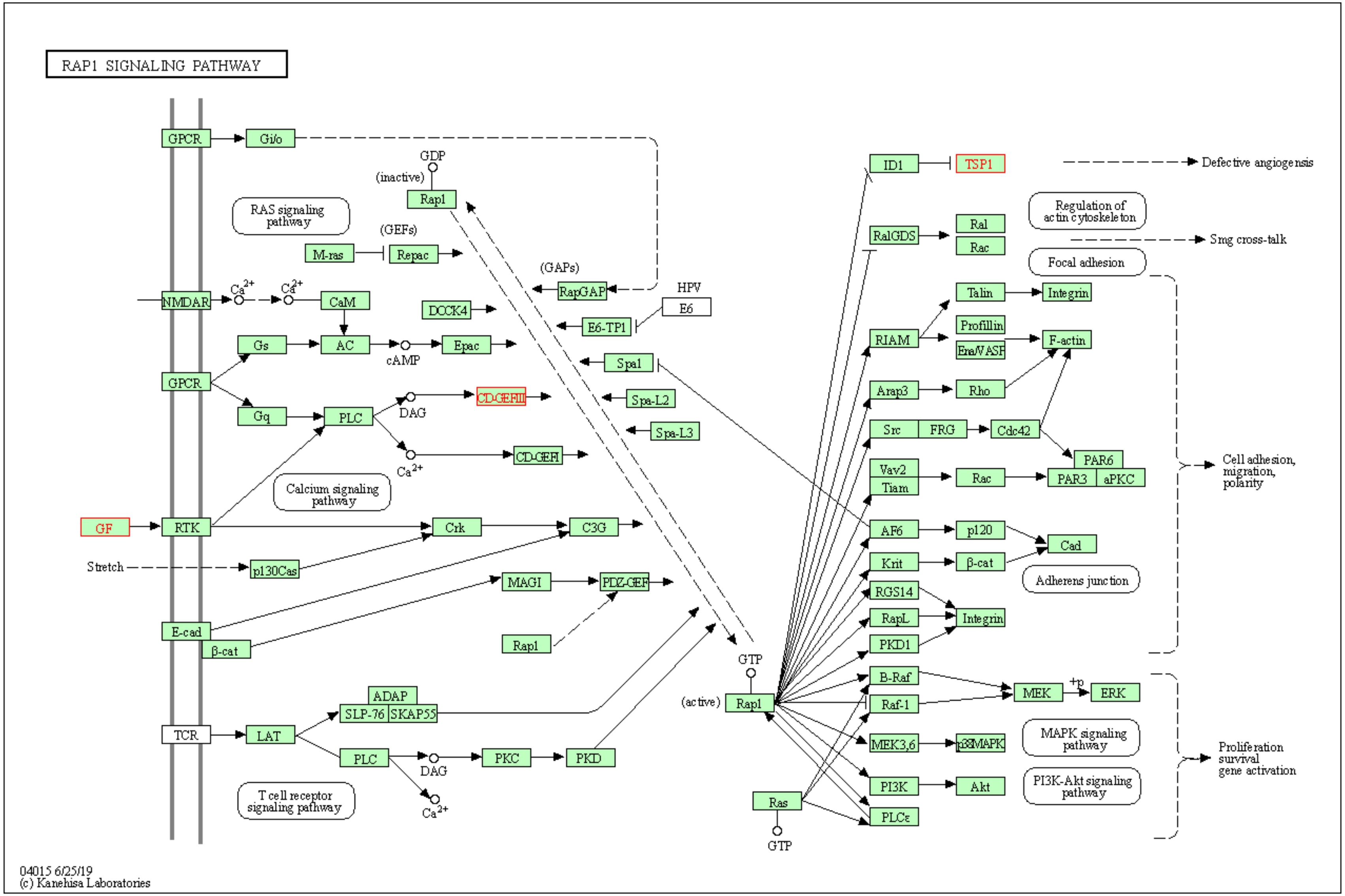

Fig.**S5**. The synergistically regulated genes mapped to the RAP1 signaling pathway

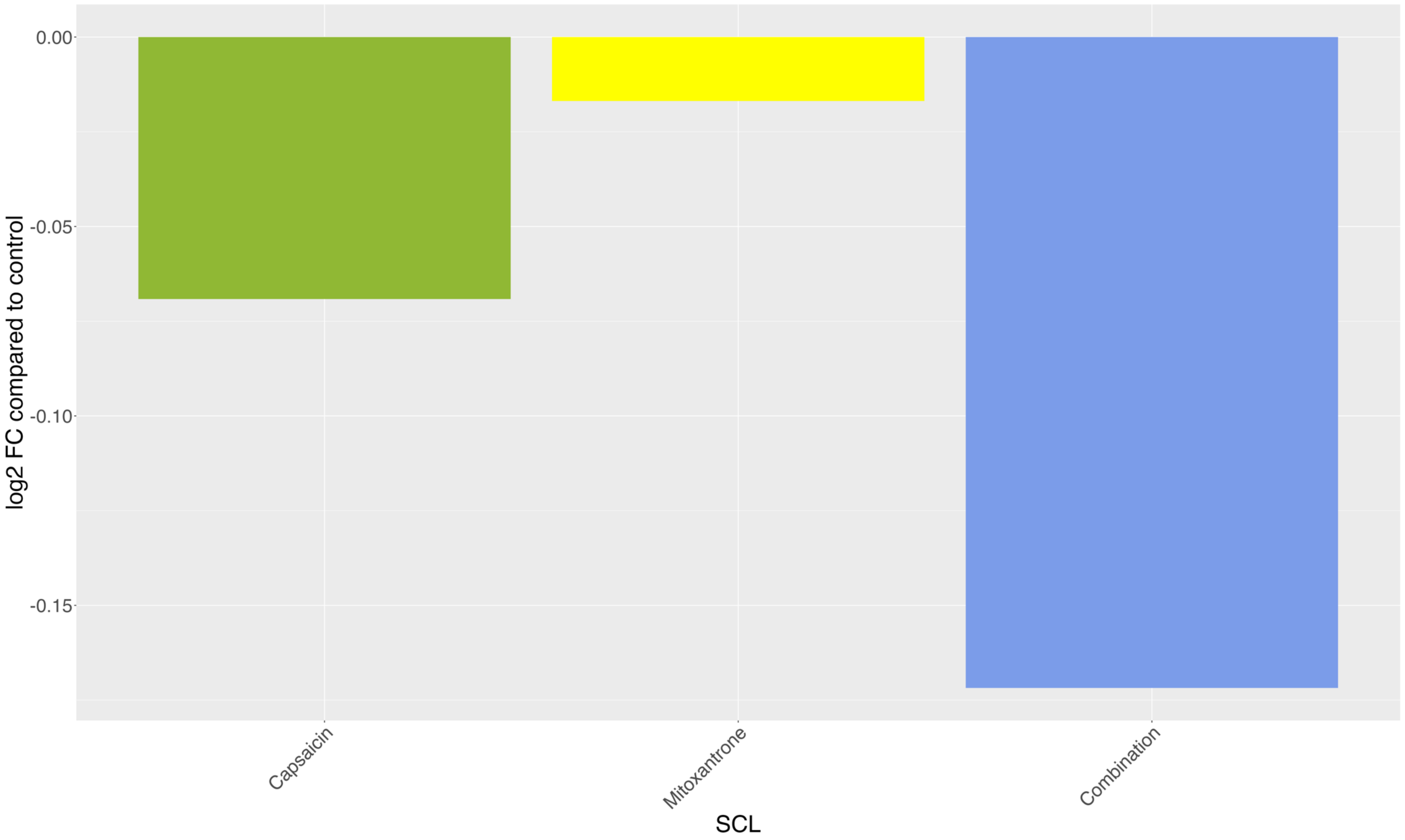

Fig.S6 The expression levels of SCL in each exposure condition
