# Supplementary material for: A network-based trans-omics approach for predicting synergistic drug combinations
Source: Commun Med (Lond). 2024 Jul 29;4:154. doi: 10.1038/s43856-024-00571-2 (PMC11286857; doi:10.1038/s43856-024-00571-2)
Supplement: Supplementary file 2 — Description of Additional Supplementary Files [file 43856_2024_571_MOESM2_ESM.pdf]

Description of Additional Supplementary Files

File name: Supplementary Data 1

File Description: The list of databases we used in this study.

File name: Supplementary Data 2

File Description: The list of genes used for constructing the human molecular interaction network from seven databases.

File name: Supplementary Data 3

File Description: The list of susceptibility genes for constructing the disease modules.

File name: Supplementary Data 4

File Description: The list of drug names and the drug response genes for constructing the drug modules.

File name: Supplementary Data 5

File Description: The list of the parameters of network propagation.

File name: Supplementary Data 6

File Description: The list of drug combinations with known synergistic effects.

File name: Supplementary Data 7

File Description: Prediction results using our proposed method.

File name: Supplementary Data 8

File Description: The list of DEGs from the microarray experiments.
